# Genetic Analysis of Apple Autumn Canopy Senescence in a Nordic Climate

**DOI:** 10.1111/ppl.70599

**Published:** 2025-10-30

**Authors:** Jonas Skytte af Sätra

**Affiliations:** ^1^ Department of Plant Breeding Swedish University of Agricultural Sciences Alnarp Sweden

**Keywords:** autumn senescence, climate adaptation, *Malus*, PBA, phenology

## Abstract

Autumn phenology traits are likely to be essential for the adaptation of apple to boreal climate. However, the genetic control of these traits is not well understood, and, for example, growth cessation does not appear to be controlled by day length as in many other boreal tree species. Here, I combine a quantitative genetic and population genomic approach to study autumn senescence in apple. I phenotyped a diverse germplasm collection for the timing of autumn senescence, performed quantitative trait loci (QTL) mapping in a multiparental population (MPP), and investigated genomic signals of selection to identify candidate genes. The timing of 50% autumn senescence was negatively correlated with adaptation to higher (boreal) climate zones. Two QTL were found to control the timing of autumn senescence in the MPP, exhibiting both dominance and epistatic interactions. The QTL on linkage group (LG) 17 was also variable in the diversity germplasm, while the QTL on LG11 was not. Cultivars adapted to boreal climate showed weak signals of selection at two loci within the genomic region of chromosome 17 corresponding to the LG17 QTL interval, consistent with a recent expansion to northern Sweden. These loci coincide with two predicted UGT85 genes and a possible copy number variation in PHYC, respectively. Thus, this study provides valuable information for further research and breeding of apple in light of the ongoing climate change.

## 1 Introduction

**FIGURE 1:**
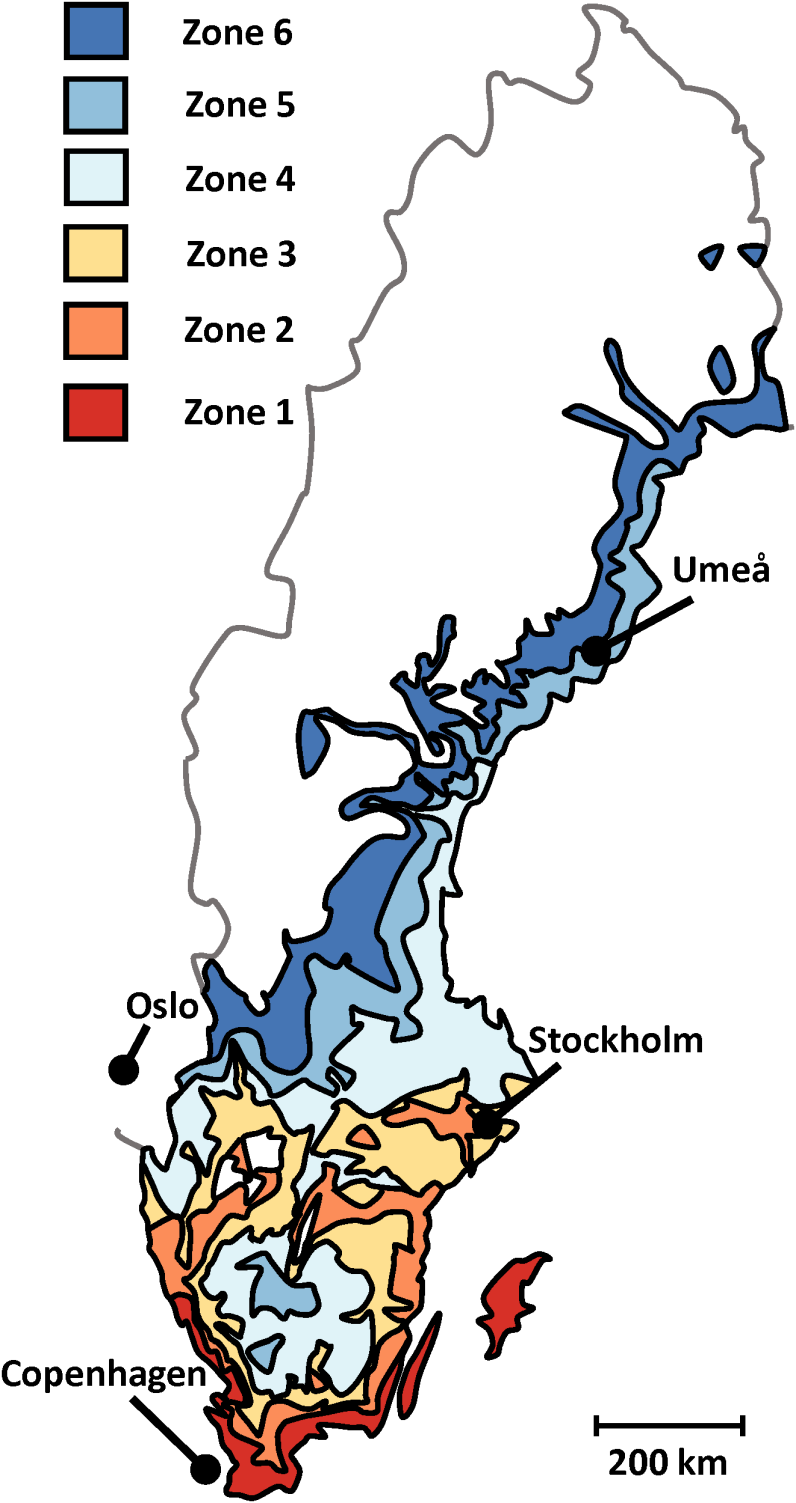
Simplified map of the Swedish horticultural climate zones 1–8, indicating Umeå and the capitals of Sweden (Stockholm), Norway (Oslo), and Denmark (Copenhagen). Climate zones 7, 8, and the alpine region are not indicated in this adaptation. Modified from the Swedish Gardens zone map (Fernqvist 1993) with permission from the National Confederation of Swedish Gardens.

Sweden is located on the Scandinavian peninsula, from 55° to 69° N, and apple ( *Malus domestica* Borkh.) is the main domestic fruit crop produced. While commercial apple production is concentrated in the southern part (56°–57° N), some commercial production exists around the capital (59° N) and some small orchards in the northern parts (65° N) (Nybom 2019). Also, temperature‐based climate models suggest that a major northward expansion of Swedish apple production could be possible by the end of the century (Meza et al. 2023). But models based on temperature alone do not account for factors such as water availability and those related to differences in latitude, such as marked changes in the solar radiation influx angle and photoperiod. Concerning the cultivation of perennial horticultural crops, Sweden is divided into nine climate zones (1–8, and the alpine region, Figure 1) (Fernqvist 1993). The system is widely used nationally and commonly used to describe the hardiness of apple cultivars in pomological literature (Näslund 2010; Nilsson 1987; Svensson 2005). For comparison, climate zones 1, 2–4, and 5–7 can be said to approximate a continental, nemoral, and boreal environmental climate zone, respectively (Metzger et al. 2005; Skytte af Sätra et al. 2024).

Adaptation of trees to temperate climate in general, and boreal climate in particular, is likely to depend on mechanisms of both fluctuating development and fixed sequence (Hänninen and Kramer 2007). Cold hardiness during winter typically follows a fluctuating development, while spring and autumn phenology follows a fixed sequence. Although spring phenology has received some attention in genetic apple research (Allard et al. 2016; Urrestarazu et al. 2017), the genetic control of the autumn syndrome (growth cessation, bud set, leaf senescence and abscission, and dormancy induction) remains largely unexplored. In *Populus* sp., one of the most well‐studied trees concerning phenology and adaptation to temperate climates, growth cessation is mainly controlled by photoperiod (Ding et al. 2024). Consequently, a main locus conferring variation in response to different daylengths has been a main target for selection among genotypes from northern Sweden (Rendón‐Anaya et al. 2021). On the other hand, growth cessation in apple has been reported to be controlled by temperature rather than photoperiod, though some studies have reported differences in vegetative extension growth under different light regimes (Garner and Allard 1923; Heide and Prestrud 2005; Hoyle 1955). There have also been seemingly conflicting reports on the effects of photoperiod on the formation of flower buds in apple (Gorter 1965, 1955).

Occurring at the later stage of the autumn syndrome, canopy autumn senescence is visually striking in many deciduous tree species and likely to reflect much of the variation in the timing of a trees preparation for winter. In apple, ectopic expression of a peach CBF gene has been reported to result in early autumn senescence, as well as increased freezing tolerance and delayed bud break (Wisniewski et al. 2015). Interestingly, the transformed apple trees were also reported to have become sensitive to photoperiod. In *Populus* , there are conflicting reports about the effect of photoperiod on autumn senescence (Fracheboud et al. 2009; Michelson et al. 2018).

Thus, the purpose of this study was to provide insights into the genetic basis for adaptation of apple to boreal climate by studying a candidate trait involved in the autumn syndrome. Toward this end I (i) assessed whether the timing of autumn senescence in a collection of heirloom apple cultivars was indeed related to adaptation to different climate zones, how consistent it was across years, and how it was impacted by temperature, (ii) identified QTL regions that segregate for differences in the timing of autumn senescence and examined associated haplotypes in the apple germplasm, and (iii) screened the identified QTL regions for evidence of selection among apple cultivars adapted to Sweden’s higher climate zones.

## 2 Materials and Methods

### 2.1 Plant Material and Phenotyping

The Swedish Central Collection of heirloom cultivars (Table 1) is located in Alnarp in southern Sweden [55.66, 13.0913] (zone 1, Supplementary File 1). It comprises over 200 accessions, each accession represented by two neighboring trees. The trees are propagated on a vigorous rootstock (‘A2’) and kept with minimal maintenance (i.e., without permanent drip irrigation, but watered in case of severe drought). For this study, I considered 216 accessions planted between 2013 and 2017, which were previously analyzed genetically (Skytte af Sätra et al. 2020) and found to consist of 185 unique genotypes. Each tree was assessed visually for percent canopy senescence (percent discolored or abscised leaves) at regular intervals (approximately every 2nd week) during the autumns of 2019, 2020, and 2021.

**TABLE 1:** Outline of genotypic datasets used in the current study.

Dataset	Composition	Phenotypic data	Genotypic data	Use
Swedish Central Collection	186 cvs.	Dense phenotyping (JD_50_, *r*)	20K SNP array (HBs)	Phenotypic and effect analysis, germplasm
Multiparental population	3 FS‐families, tot. 496 ind.	Sparse phenotyping (%SEN)	20K SNP array (pruned SNPs)	QTL analysis
Selection scan	59 cvs.	—	Re‐sequencing	Scan for signals of selection

The multiparental population (MPP) consists of three pedigree‐connected full‐sibling (FS) families (Table 1), comprising diploid offspring from the crosses ‘Aroma’ × ‘Discovery’ (ArDi, *n* = 172), ‘Santana’×‘Katja’ (SaKa, *n* = 177), and ‘Santana’ × ‘Aroma’ (SaAr, *n* = 147). The pollen parent of the ‘Santana’ × ‘Aroma’ family was actually ‘Amorosa’, a red‐skinned sport of ‘Aroma’, which for the purpose of this work is considered genetically identical to ‘Aroma’. The crosses were made in 2012, planted on own roots in a field at Balsgård in southern Sweden [56.1056, 14.1797] (zone 1, Supplementary File 1) in the autumn of 2014, and have been kept with limited maintenance since then. A simplified procedure was used to phenotype the MPP in 2022 and 2023, based on experiences from the Swedish Central Collection. As the largest differences between genotypes were seen in October, the MPP was assessed visually for percent canopy senescence on three dates every second week around that time, that is, September 30, October 13, and October 27 in 2022, and October 2, October 16, and November 1 in 2023. The observations were summarized as average % senescent canopy across all six observations (%SEN), which was used as trait parameter for analysis of the MPP. Five trees at the eastern border of each row were excluded due to clear border effects (Supplementary File 1). Trees that could not be assessed reliably, due to, for example, abnormal growth behavior or signs of stress, were also excluded. This left 150, 166, and 137 individuals with phenotypic data from the ArDi, SaKa, and SaAr FS families, respectively.

The set of apple cultivars used for scans of local signals of selection (Table 1) was identified and analyzed in a previous study (Skytte af Sätra et al. 2024). Briefly, Swedish pomological literature was reviewed (Näslund 2010; Nilsson 1987; Svensson 2005), and 59 apple cultivars were selected based on their highest recommended climate zones. Thus, 29 cultivars were designated as ‘not hardy’, recommended up to zones 1 or 2, and 30 cultivars were designated as ‘hardy’, being recommended up to zones 5 or 6. Of these, 18 and 23 cultivars were present in the Swedish Central collection (Supplementary File 2), respectively.

### 2.2 Genotypic Data

Raw intensity data from the 20K Infinium SNP array (Illumina Inc.) (Bianco et al. 2014) was available for genotypes from the Swedish Central Collection, the MPP, and a wider associated pedigreed germplasm from previous and ongoing research projects (Skytte af Sätra, Odilbekov, et al. 2023; Skytte af Sätra 2023; Skytte af Sätra et al. 2020; Skytte af Sätra and Garkava‐Gustavsson 2022). Genotypic data was curated as described by Vanderzande et al. (2019), with some modifications. Initially, a subset of 10K SNPs from a study on marker integration (Howard et al. 2021) was retained, and genotypes were called in Genome Studio (GS) v2.0 (Illumina Inc.), using cluster definitions kindly provided by the authors. For computational efficiency, markers for QTL analysis in the MPP were pruned for pairwise *r* ^2^ above 0.8 in 2 Mb sliding windows with a 1 SNP shift using PLINK 1.9 (Chang et al. 2015) and physical positions on the HFTH1 whole‐genome sequence (WGS) (Skytte af Sätra, Larsen, et al. 2023; Zhang et al. 2019). Following the curation of Mendelian‐inconsistent and Mendelian‐consistent errors, this resulted in 5248 SNPs used for QTL analysis of the MPP. Subsequently, a full set of markers mapped to linkage group (LG) 11 and LG17 was curated (Vanderzande et al. 2019). Briefly, SNP calls were curated considering Mendelian‐inconsistent and Mendelian‐consistent errors, HBs were defined using FlexQTL and PediHaplotyper (Voorrips et al. 2016), and HB calls were curated considering Mendelian‐inconsistent and Mendelian‐consistent errors. Thus, 657 and 518 SNPs targeting LG11 and LG17 were summarized into 134 and 108 HBs, respectively, which were used for analysis of haplotype effects in the MPP and the germplasm analysis.

For curation and analysis, genetic positions were taken from an advanced draft of a virtual linkage map (the iGW‐map), resulting from the integration of the iGLMap and the HFTH1 WGS (Di Pierro et al. 2016; Skytte af Sätra, Larsen, et al. 2023; Zhang et al. 2019).

Whole‐genome re‐sequencing data of 59 apple cultivars was available from previous work (Skytte af Sätra et al. 2024). Briefly, cultivars were sequenced with 150 bp pair‐end reads to an average depth of ~24× per haploid genome and aligned against the HFTH1v1 reference genome (Zhang et al. 2019), resulting in 17.3 M biallelic SNPs after calling and filtering.

Genotypes at the S‐locus were taken from literature (Broothaerts and Van Nerum 2003; Larsen et al. 2016; Nybom et al. 2008), assuming all cultivar samples to be true‐to‐type, and in the case of ‘Santana’, deduced from haplotype transmission. The approximate HFTH1 position of the S‐locus was estimated by a BLAST of the EIIWPN‐R/ASPF3‐F primer‐pair sequences described for S‐locus genotyping (Larsen et al. 2016).

### 2.3 Phenotypic Analysis of Germplasm

The development of autumn canopy senescence for each tree in the Swedish Central Collection, each season, was modeled by fitting a logistic function as implemented in Growthcurver (Sprouffske and Wagner 2016):

(1) $$N_{t}=\frac{K}{1+\left(\frac{K-N_{0}}{N_{0}}\right)e^{-r_{sen}t}}$$

where *N*_*t*_ is the senescence percentage of the canopy at time *t*, *K* is the carrying capacity (maximum percent canopy senescence in the model), *N*_0_ is the percentage of canopy senescence at the beginning of the model, and *r*_*sen*_ is the maximal intrinsic growth rate of senescence which would occur if there were no restrictions. In addition to the actual assessments, two data points were added post hoc to each tree and season to anchor the regression: the first of August as 0% senescence and the last of December as 100% senescence. Then, the calendar dates relative to August 1st at which 50% canopy senescence was reached according to the model (JD_50_) were interpolated for each tree in each season.

To assess year‐to‐year correlations, the following mixed model was fitted to the data from each year separately:

(2) $$P_{\mathrm{ijk}}=\mu +\Gamma_{i}+{\mathrm{YP}}_{j}+e_{\mathrm{ijk}}$$

where *P*_*ijk*_ is the value of the phenotypic response variable (JD_50_ or *r*) of the *i*‐th genotype for the *j*‐th year of planting and the *k*‐th tree, *μ* is the model intercept, *Γ*_*i*_ is the fixed effect of the *i*‐th genotype, *YP*_*j*_ is the random effect of the *j*‐th year of plantation, and *e*_*ijk*_ is the residual of the model. The correlations of best linear unbiased estimates (BLUEs) of genotype effects between years were evaluated by Pearson’s product–moment correlation.

Point estimates of broad sense heritability (*Ĥ* ^2^) across all 3 years of phenotyping were calculated by regressing best linear unbiased predictors (BLUPs) on BLUEs, whereby the regression coefficient can be interpreted as an approximation of *Ĥ* ^2^ (Schmidt et al. 2019). Genotype BLUEs (Equation 3) and BLUPs (Equation 4) across years were estimated using the following models:

(3) $$P_{\text{ijklm}}=\mu +\Gamma_{i}+Y_{j}+{\mathrm{YP}}_{k\left(j\right)}+A_{l\left(k\left(j\right)\right)}+e_{\text{ijklm}}$$

(4) $$P_{\text{ijklm}}=\mu +G_{i}+Y_{j}+{\mathrm{YP}}_{k\left(j\right)}+A_{l\left(k\left(j\right)\right)}+e_{\text{ijklm}}$$

where *P*_*ijklm*_ is the value of the phenotypic response variable (JD_50_ or *r*) of the *i*‐th genotype for the *j*‐th year of phenotyping of the *k*‐th year of planting nested within year of phenotyping of the *l*‐th accession nested within year of planting and the *m*‐th tree, *μ* is the model intercept, *Γ*_*i*_ is the fixed effect of the *i*‐th genotype, *G*_*i*_ is the random effect of the *i*‐th genotype, *Y*_*j*_ is the random effect of the *j*‐th year of phenotyping, *YP*_*k*(*j*)_ is the random effect of the *k*‐th year of plantation nested within the *j*‐th year of phenotyping, *A*_*l*(*k*(*j*))_ is the random effect of the *l*‐th genotypic replicate (some genotypes were duplicated within the collection), and *e*_*ijklm*_ is the residual.

Year effects were estimated using the following model:

(5) $$P_{\text{ijkl}}=\mu +\Upsilon_{i}+G_{j}+{\mathrm{YP}}_{k\left(j\right)}+e_{\text{ijkl}}$$

where *P*_*ijkl*_ is the value of the phenotypic response variable (JD_50_ or *r*) for the *i*‐th year of assessment of the *j*‐th genotype of the *k*‐th year of planting of the *l*‐th tree, *μ* is the model intercept, *Υ*_*i*_ is the fixed effect of the *i*‐th year of assessment, *G*_*j*_ is the random effect of the *j*‐th genotype, *YP*_*k*(*j*)_ is the random effect of the *k*‐th year of plantation nested within genotype effect, and *e*_*ijkl*_ is the residual.

Different models for cumulative cooling day degrees (CDDs) were used to assess the impact of variation in temperature on the timing of JD_50_ in different years. CDDs were estimated as the difference between the daily mean temperature and the respective base values, accumulated from August 1 until JD_50_ for the intercept plus the year effect estimated from Equation (2). Models with bases 30°C, 25°C, 20°C, 15°C, 10°C, and 5°C were considered. Daily mean values equal to or exceeding the base values were treated as 0 CDDs. Meteorological data was collected from the Lönnstorp field station (Lönnstorp Research Station 2022, 2021a, 2021b), located approximately 1.4 km from the Swedish Central collection.

The three main recent Swedish pomologies were reviewed (Näslund 2010; Nilsson 1987; Svensson 2005) for recommendations on the highest horticultural climate zone (Fernqvist 1993) for the apple cultivars in the Swedish Central Collection. The correlation between the highest recommended zone and BLUEs for JD_50_ across years (Equation 3) was evaluated by both Pearsons product–moment correlation and Stuarts tau‐*c*. Differences in variance ratios for JD_50_ between genotype groups were compared by F‐test. The phenotypic data from the Swedish Central collection have previously been described and analyzed briefly in a conference proceeding and a doctoral thesis (Skytte af Sätra, Hjalmarsson, et al. 2023; Skytte af Sätra 2023).

### 2.4 QTL Analysis

QTL mapping in the MPP was performed using a Bayesian approach embedded in FlexQTL (www.flexqtl.nl), using Markov chain Monte Carlo (MCMC) simulations and bi‐allelic QTL models (Bink et al. 2014, 2008). Four independent FlexQTL runs were performed with %SEN as phenotype, using different seeds and priors for the number of QTL (1 or 3) considering a maximum of six QTL. All runs consisted of 100,000 iterations with a thinning of 100 using additive genetic models with normal prior distributions and random (Co) variance matrix diagonals. Marker segregation distortion was allowed (MSegDelta = 1) and singletons were excluded (DeleteDR = 1). Phenotypes of parents were not included in the analysis. FlexQTL indicates the level of evidence provided for the presence of a QTL as two times the natural logarithm of the Bayes factors (“2lnBF”) for an incremental number of QTLs per LG, through a pair‐wise comparison. A 2lnBF value above 0, 2, 5, and 10 is considered to indicate ‘hardly any’, ‘positive’, ‘strong’, and ‘decisive’ evidence for the presence of a given number of QTL, respectively. QTL intervals constitute successive 2 cM bins with 2lnBF above 2. FlexQTL infers QTL genotypes, denoting increasing (early senescence) and decreasing (late senescence) alleles as ‘*Q*’ and ‘*q*’, respectively, and this notation is used throughout this paper. QTL trace plots and posterior positions were visualized by a set of R‐scripts accompanying VisualFlexQTL, the latter being modified to compile all four runs in a single plot. Identity‐by‐Descent (IBD) probabilities for LG11 and 17 were estimated in FlexQTL and visualized in PediMap (Voorrips et al. 2012).

For analysis of haplotype effects in the MPP, phased HB marker data was used considering the ‘outer QTL interval’, that is, the lowest start position and the highest end position for the QTL interval across the four replicate runs of FlexQTL. Only individuals without recombination events or missing value genotypes within the ‘outer QTL interval’ were retained. The Kolmogorov–Smirnov test, implemented in the ‘stats’ package (R Core Team 2020), was used to test the statistical significance of phenotypic distributions between genotypes, inspired by previous work (Van de Weg et al. 2018). Thus, phenotypic distributions were illustrated as empirical cumulative distribution (ECDF) plots, using ggplot2 (Wickham 2016).

### 2.5 Germplasm Effects

A two‐stage approach was used to analyze the potential haplotype effects of QTL intervals in the Swedish Central Collection, utilizing the characteristics of genotypic data from the apple 20K SNP array (Bianco et al. 2014). The 20K SNP array was developed based on a focal‐point design, whereby the SNPs are clustered in narrow genomic regions, up to 11 SNPs within 10 kb according to the draft apple WGS GDv1.0 (Velasco et al. 2010). During the conversion of individual SNP markers to virtual HB markers, HBs will largely represent these tightly linked focal points. Consequently, most of the apple genome is located between pairs of HBs, rather than in linkage disequilibrium (LD) with individual markers. Through the current data curation process (Vanderzande et al. 2019), marker data is fully linkage‐phased through the use of pedigree information, such that HBs represent consecutive haplotypes along each homolog. Realizing this, a series of consecutive HBs (2–30) across the two chromosomes were analyzed with lme4QTL (Ziyatdinov et al. 2018) using the following model:

(6) $$P_{\mathrm{ijk}}=\mu +{Η}_{i}+G_{j}+e_{\mathrm{ijk}}$$

where *P*_*ijk*_ is the value of the phenotypic response variable (BLUE for JD_50_ from Equation 2) for the *i*‐th joint identical‐by‐state (IBS) haplotype of the *j*‐th genotype of the *k*‐th year of phenotyping, *μ* is the model intercept, *Η*_*i*_ is the fixed effect of the *i*‐th joint haplotype, *G*_*j*_ is the random effect of the *j*‐th genotype, and *e*_*ijk*_ is the residual. Additionally, a G‐matrix estimated with AGHmatrix (Amadeu et al. 2016; VanRaden 2008) from the genotypes genome‐wide 20K array SNP marker data was used as a covariance matrix. Both haplotypes of each genotype were assigned the same phenotypic value and only haplotypes with a minor allele frequency (MAF) above 0.05 were considered. To penalize very long haplotypes, with very few individuals retained, models were evaluated by a simple index (pIndex) by multiplying the proportion of individuals retained after filtering for MAF with the negative log_10_‐transformed *p* value for the haplotype effect in the model. Haplotypes for consideration were required to overlap with the genomic regions corresponding to the inner QTL intervals, while not having a length exceeding that of the outer QTL interval of the respective QTL. The haplotype that maximized the pIndex while meeting these conditions was considered the most sensible option and was further analyzed.

Next, the effects (increasing/decreasing) of haplotypes with MAF above 0.05 in the Swedish Central Collection were identified by post hoc tests. First, individual joint IBS HB haplotypes were evaluated in a linear model with BLUEs for JD_50_ across years (from Equation 3) as the response variable, and the heteroscedasticity and normality of the residuals of the model were evaluated by a studentized Breusch–Pagan test and a Shapiro–Wilk normality test, respectively (R Core Team 2020; Zeileis and Hothorn 2002). Then, Duncans new multiple range test as implemented in the agricolae package (De Mendiburu 2009) was performed, with a relaxed alpha of 0.1.

### 2.6 Local Signals of Selection

Pairwise *F*_ST_, Tajima’s *D*, and nucleotide diversity (π) were estimated in 10 kb windows using VCFtools (Danecek et al. 2011), as previously described (Skytte af Sätra et al. 2024). Predicted genes in the 9.5–11.5 Mb region of chromosome 17 of the HFTH1v1 WGS and annotations of corresponding transcripts were retrieved from the Genome Database for Rosaceae (GDR) (Jung et al. 2018) [accessed 2024‐12‐17]. Similarly, predicted genes with a functional annotation containing “phytochrome” and either of “PHYA”, “PHYB”, “PHYC”, or “PHYE” in the BLAST field were retrieved from the GDDH13v1.1 (Daccord et al. 2017), HFTH1 (Zhang et al. 2019), Gala diploid (Sun et al. 2020), Malus sylvestris diploid (Sun et al. 2020), Malus sieversii diploid (Sun et al. 2020), Honeycrisp (Khan et al. 2022), Antonovka 172,670‐B (Švara et al. 2023), WA‐38 (Zhang et al. 2024), Fuji (Li et al. 2024), M9 (Li et al. 2024), and MM106 (Li et al. 2024) WGS assemblies through the GDR [accessed 2025‐04‐29].

## 3 Results

### 3.1 Autumn Senescence in the Swedish Central Collection of Heirloom Cultivars

**FIGURE 2:**
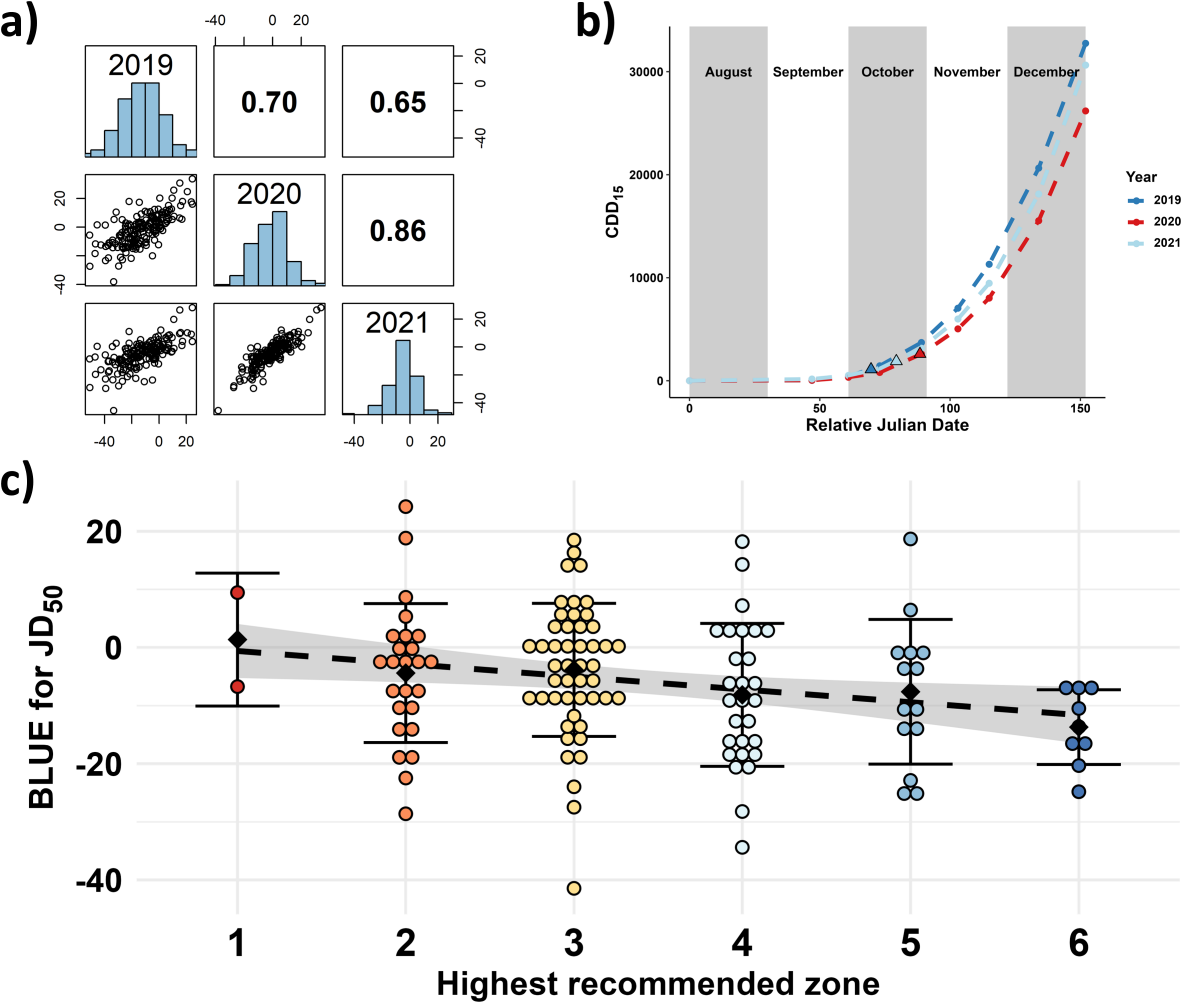
Date of 50% canopy autumn senescence relative to August 1st (JD_50_), (a) correlogram of genotype BLUEs for JD_50_ between 3 years with Pearson’s correlation coefficients in the upper‐right off‐diagonal, (b) accumulated CDDs (base 15°C) for 3 years with the BLUEs for year effect plus intercept indicated by triangles, and (c) the correlation between JD_50_ (*y*‐axis) and highest recommended climate zone for cultivation from pomological literature (*x*‐axis, fill color as in Figure 1) for a subset of cultivars in the Swedish Central collection. Black diamonds indicate group mean, error bars +/− one standard deviation, the black‐dotted line represents the linear relationship between the parameters, with the gray‐shaded area indicating the 95% confidence interval of the curve.

First, I assessed autumn canopy senescence in the Swedish Central Collection of heirloom cultivars over 3 years. The date of 50% canopy autumn senescence (JD_50_) in the Swedish Central Collection of heirloom cultivars showed strong to very strong correlations between the 3 years (*r* = 0.65, 0.70 and 0.86; Figure 2a), and were all highly significant (*p* < 0.001). Accordingly, JD_50_ had a high point estimate for broad‐sense heritability across years (*Ĥ* ^2^ = 0.81) in the collection. Nevertheless, year effects were highly significant (*p* < 0.001), with 2020 and 2021 having fixed effects of 18.8 and 9.7 days, respectively. Thus, I investigated the impact of autumn temperature on autumn senescence. Although all considered CDD models had strong or very strong correlations with the Year‐effect for JD_50_, only the CDD_15_ model had a statistically significant correlation (*p* < 0.001, *r* = 1.00, Figure 2b and Supplementary File 1). At last, considering a subset of 126 genotypes for which recommended climate zones were available in pomological literature, the highest recommended climate zone was significantly correlated with JD_50_ (*r* = −0.22; *p* = 0.01) (tau‐*c*: −0.17; CI_99%_ −0.34 to −0.005). Thus, the cultivars recommended only up to zone 1 reached 50% canopy autumn senescence on average 15 days later than those recommended up to zone 6 (Figure 2c). Additionally, cultivars recommended for cultivation up to zone 6 exhibited a lower variance for JD_50_ than the other cultivars combined (ratio = 0.29; *p*_less_ = 0.03), indicating directional selection.

On the other hand, the theoretical maximal rate of senescence (*r_sen_*) was not significantly correlated with the cultivar’s highest recommended climate zone (*r* = −0.08, *p* > 0.05) (tau‐*c*: −0.06; CI_95%_ −0.20 to 0.07). This parameter exhibited a low heritability in the collection (*Ĥ* ^2^ = 0.38), and low correlations between years (*r* ≤ 0.3), though significant between 2020 and 2021 (*p* < 0.01). Year effects were highly significant (*p* < 0.001), but none of the CDD models were significantly correlated with the year effects of this parameter. At last, JD_50_ was very weakly correlated with *r*_sen_ (*r* = 0.12, *p* < 0.001, Supplementary File 1), indicating that only a very small proportion of the variation for JD_50_ was due to the rate of senescence.

### 3.2 Multiparental QTL Mapping

**FIGURE 3:**
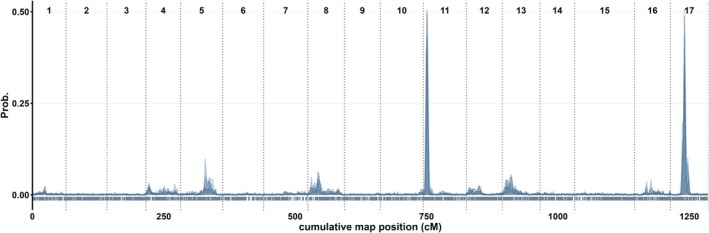
Summary of posterior positions from four FlexQTL runs, with each replicate represented by a semi‐transparent layer.

Next, I used a MPP representing the key germplasm for modern Swedish apple breeding to map QTL for canopy autumn senescence. The parents of the MPP are recommended up to zones 2–3, thereby being more likely to segregate for loci associated with autumn canopy senescence than more hardy cultivars, given the indication of directional selection. Accordingly, I found decisive evidence for 2 QTL (Figure 3), one on LG11 (2lnBF: 10.8–13.5) and one on LG17 (2lnBF: 31.0–33.3). There was no strong evidence (2lnBF > 5) for any other QTL, on the same LG or on another LG, for any replicate run (Supplementary File 2). Both QTL intervals were reproducible across runs, and all replicate runs converged (ECS > 100 for all parameters). The two QTL were discovered independently in all replicate runs, according to the trace plots (Supplementary File 1). The QTL intervals of all four replicate runs were investigated, confirming the presence of segregating markers and recombination events. Inference of parental genotypes was consistent across all four runs, with strong evidence in all cases except for two parents that had only positive evidence in one run each (Table 2). The locus on LG11 had an average mode of 7.25 cM_iGW_ and included a 5–10 cM_iGW_ region in all replicate runs (‘inner’ QTL interval). The lowest start and highest end positions across the four runs spanned 2–15 cM_iGW_ (‘outer’ QTL interval). The locus on LG17 had an average mode of 27.25 cM_iGW_ and included a 25–34 cM_iGW_ region in all replicate runs (‘inner’ QTL interval). The lowest start and highest end positions across the four runs spanned 21–38 cM_iGW_ (‘outer’ QTL interval) for the LG17 locus.

**TABLE 2:** Summary of QTL intervals with decisive evidence across four replicate FlexQTL runs.

QTL	Pos.	Start		Mode	End		Parent genotypes			
		Min	Max	Mean	Min	Max	‘Aroma’	‘Discovery’	‘Katja’	‘Santana’
LG11	Gen.	2.0	5.0	7.3	10.0	15.0	*Qq*	*QQ* ^a^	*QQ*	*QQ*
	Phys.	0.9	2.1	3.1	4.3	6.4				
LG17	Gen.	21.0	25.0	27.3	34.0	38.0	*Qq* ^a^	*Qq*	*Qq*	*Qq*
	Phys.	7.1	8.5	9.3	11.7	13.3				

*Note:* For each of the two QTL, the genetic (Gen., cM_iGW_) and physical (Phys., Mb_HFTH1_) position of the lowest and highest start and stop of the QTL interval across all four runs is indicated, as well as the average QTL mode. Genotypes of the four parents are noted, as inferred through FlexQTL.

^a^ Two inferred genotypes had positive evidence in one of the four runs, and strong evidence in the other three runs. All other genotypes had strong evidence across all four runs.

**FIGURE 4:**
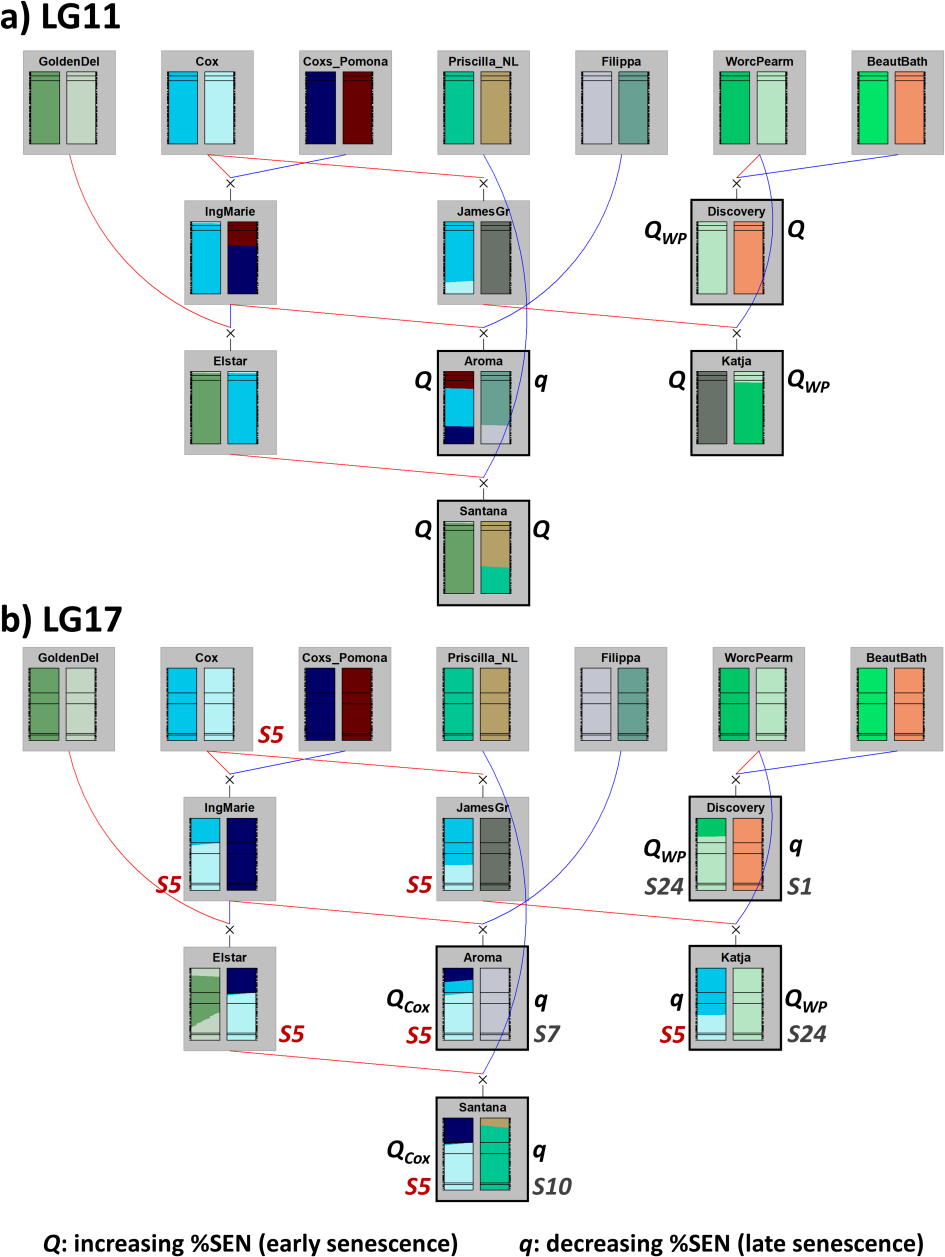
Haplotype transmission among progenitors of the MPP, for (a) LG11 and (b) LG17. For each individual, the two bars indicate the probabilities of being IBD with the founder haplotypes. The left bar indicates the maternal homolog, with inheritance indicated by red lines, and the right bar indicates the paternal homolog, with inheritance indicated by blue lines. Parents of the MPP are indicated with thick frames. The mapped QTL are indicated by horizontal lines, indicating boundary markers for the inner intervals. The approximate position of the S‐locus is indicated by two horizontal lines corresponding to markers flanking the approximate position of the locus. QTL effects (*Q*/*q*) are indicated adjacent to each parent, specifying alleles that are IBD in two parents (*Q*_WP_ and *Q*_Cox_). Note that the *Q*_WP_ allele is IBS with the *Q*_Cox_ allele within the inner QTL interval (Supporting Information 2). Similarly, S‐alleles of each parent of the MPP are indicated, with the S5 allele in red and traced through the pedigree. For some cultivars, abbreviated names are used, see Supporting Information 2 for accepted cultivar names.

Despite being unselected, two of the FS families seemed to exhibit segregation distortion for the paternal haplotypes of the QTL at LG17. Reviewing the reported and deduced genotypes of the parents at the S‐locus revealed that in the FS‐families ‘Santana’ × ‘Katja’ and ‘Santana’ × ‘Aroma’, both parents had one S‐allele in common (Figure 4). Investigating the allele frequencies of individual SNP markers in the FS‐families revealed a linear decline in segregating distortion away from the S‐locus on LG17 (Supplementary File 1). Thus, the segregation distortion is likely caused by the S‐locus, rather than being, for example, an artifact caused by another locus, lethality at the segregating locus, or pre‐selection. Although segregation distortion can affect the power to identify QTL, a sufficient number of individuals (> 20) inherited the haplotypes in coupling phase with the paternal *S5*‐allele in both FS families to allow meaningful further analysis.

### 3.3 QTL Effects in Segregating Families

**FIGURE 5:**
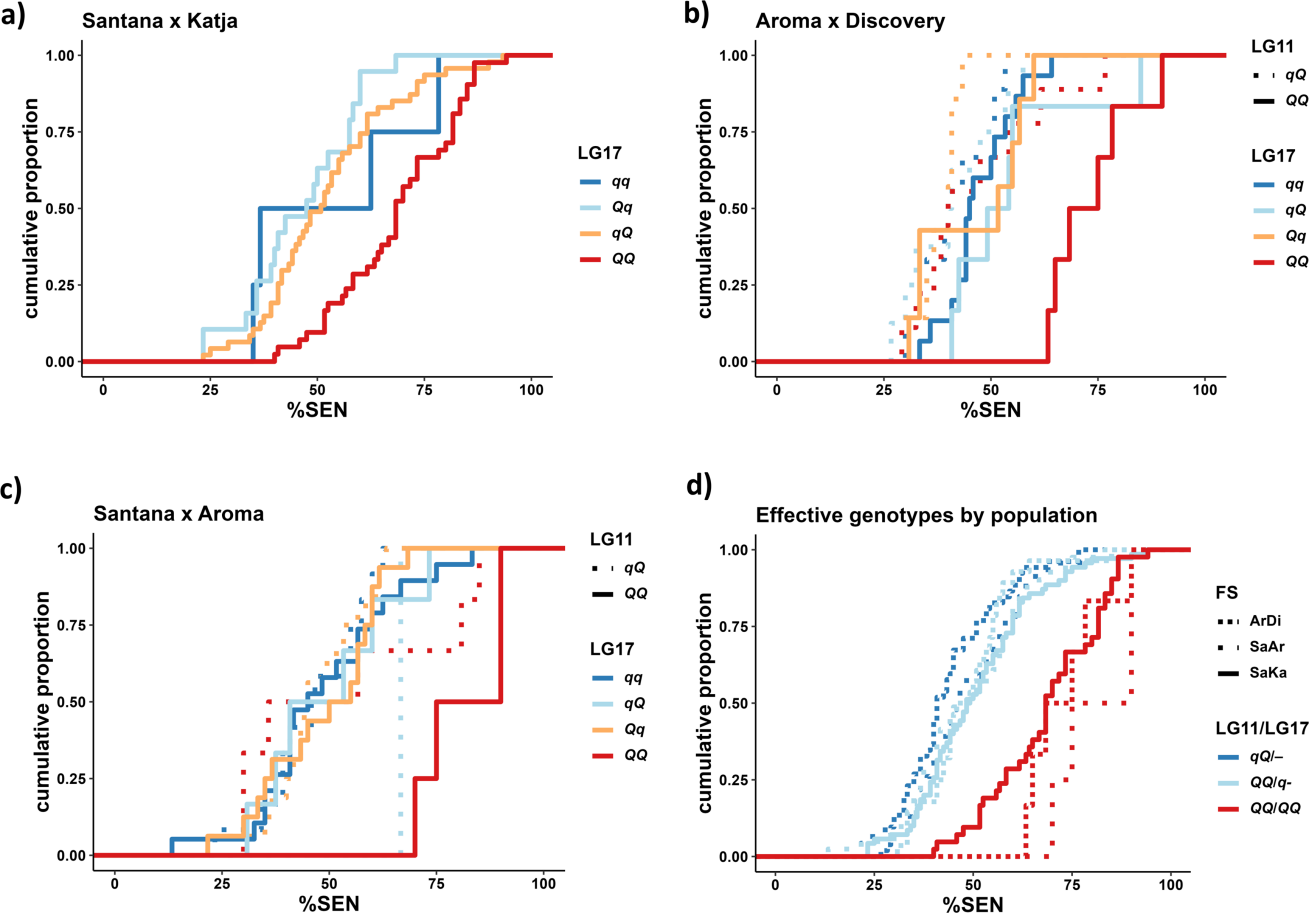
Cumulative distributions of mean % senescence (%SEN) in (a) ‘Santana’ × ‘Katja’ (SaKa), (b) ‘Aroma’ × ‘Discovery’ (ArDi), (c) ‘Santana’ × ‘Aroma’ (SaAr), and (d) effective compound genotypes by FS‐family.

Next, I investigated the allelic effects and possible interactions within and between the mapped QTL. The ‘Santana’ × ‘Katja’ FS‐family segregates for only the LG17 locus. ‘Santana’ has inherited one *q* allele from ‘Priscilla‐NL’, and ‘Katja’ has inherited one *q* allele from ‘James Grieve’, and the family exhibits single locus dihybrid segregation. The two heterozygote groups were not significantly different from one another (*p* = 0.51), and both heterozygotes were significantly different from the homozygous *QQ* genotype (*p* < 0.0001). The homozygous *qq* was not significantly different from either of the other genotype groups, though, most likely because of the very small sample size of this genotype group (*n* = 4). However, the distribution curve of the *qq* genotype group is relatively close to those of the two heterozygotes, and the group mean is very close to the *qQ* group (53.1 %SEN vs. 52.1 %SEN). Thus, the *q* allele (late senescence) seems to be dominant over the *Q* allele (early senescence, Figure 5a).

The ‘Aroma’ × ‘Discovery’ FS‐family segregates for both the LG11 and the LG17 loci and does not exhibit segregation distortion at the LG17 locus. Only ‘Aroma’ segregates for the LG11 locus, with a *q* allele coming from ‘Filippa’, whereas ‘Discovery’ is inferred as homozygous *QQ* by FlexQTL. Both parents segregate for the LG17 locus, and ‘Aroma’ has inherited a *q* allele from ‘Filippa’, whereas ‘Discovery’ has inherited a *q* allele from ‘Beauty of Bath’. Considering the individuals that are homozygous *QQ* at the LG11 locus, the LG17 locus exhibits the same dominance pattern as in ‘Santana’ × ‘Katja’. The two heterozygotes were not significantly different from one another (*p* = 0.43), and they were not significantly different from the homozygous *qq* either (*p* = 0.46 and 0.71). On the other hand, the homozygous *QQ* group was significantly different from the homozygous *qq* (*p* < 0.001) and the two heterozygotes (*p* < 0.05 and 0.01). However, considering the individuals that are heterozygous *qQ* at the LG11 locus, none of the comparisons corresponding to the above were significant (*p* ≥ 0.30). Thus, the two loci exhibit both dominance and epistasis in this family, such that only individuals being homozygous *QQ* at both loci exhibit the early senescence phenotype (Figure 5b).

The ‘Santana’ × ‘Aroma’ FS‐family segregates for both the LG11 and the LG17 loci, with allele origins as above, and exhibits segregation distortion at the LG17. Considering the individuals that are homozygous *QQ* at the LG11 locus, the LG17 locus exhibits the same dominance pattern as the other families. The two heterozygotes were not significantly different from one another (*p* = 0.98), and they were not significantly different from the homozygous *qq* either (*p* ≥ 0.90). On the other hand, the homozygous *QQ* group was significantly different from the homozygous *qq* (*p* < 0.01), and the two heterozygotes (*p* < 0.05 and 0.01). However, considering the individuals that are heterozygous *qQ* at the LG11 locus, none of the comparisons corresponding to the above were significant (*p* > 0.10), though one of the double heterozygotes (*qQ*/*qQ*) was only represented by a single individual. Thus, the two loci exhibit both dominance and epistasis in this family (Figure 5c), similar to ‘Aroma’ × ‘Discovery’.

Finally, the three effective compound genotypes (*qQ*/‐‐, *QQ*/*q*‐, and *QQ*/*QQ*) were not significantly different between the three FS families (*p* > 0.1), indicating that they have the same effects across the current genetic backgrounds (Figure 5d).

To search for potential additional epistatic loci, the MPP was split into three groups by effective compound genotypes for the LG11 and LG17 loci. A single FlexQTL run was performed using subsets of individuals being *qQ*/‐‐ (*n* = 98), *QQ*/*q*‐ (*n* = 139), or *QQ*/*QQ* (*n* = 49) for the LG11 and LG17 loci. The *qQ/*‐‐ group showed positive evidence (2lnBF_1/0_: 4.4) for a QTL on LG9 (interval: 2–50 cM, mode: 18 cM). The *QQ*/*q*‐ group showed positive evidence for one QTL (2lnBF_1/0_: 2.1) on LG10 (interval: 1–30 cM, mode: 27 cM), and one QTL (2lnBF_1/0_: 2.1) on LG13 (interval: 1–38 cM, mode: 17 cM). The *QQ*/*QQ* group did not show positive evidence for any QTL. There was no strong or decisive evidence for any QTL in any of the groups. Being the epistatic candidate locus with the highest power, approaching the threshold for strong evidence for a QTL (2lnBF_1/0_ > 5), the LG9 locus was investigated briefly. Thus, the single FlexQTL run indicated strong evidence for ‘Santana’ being *Qq*, and inconclusive genotype assignments for ‘Aroma’ and ‘Discovery’. One parent of ‘Santana’, ‘Priscilla‐NL’, had positive evidence for being *qq*. Although genotype assignments for the other parent were inconclusive, this indicates that ‘Santana’ likely inherited the *q* allele from ‘Priscilla‐NL’ and the *Q* allele from ‘Elstar’. The presence of segregating markers within the QTL interval was also confirmed for ‘Santana’. Although these provisional results should be treated with caution, due to the small sample sizes and low statistical power, they indicate that there might be additional loci segregating in the germplasm, involved in further epistatic interactions with the loci described here.

### 3.4 Germplasm Effect

**FIGURE 6:**
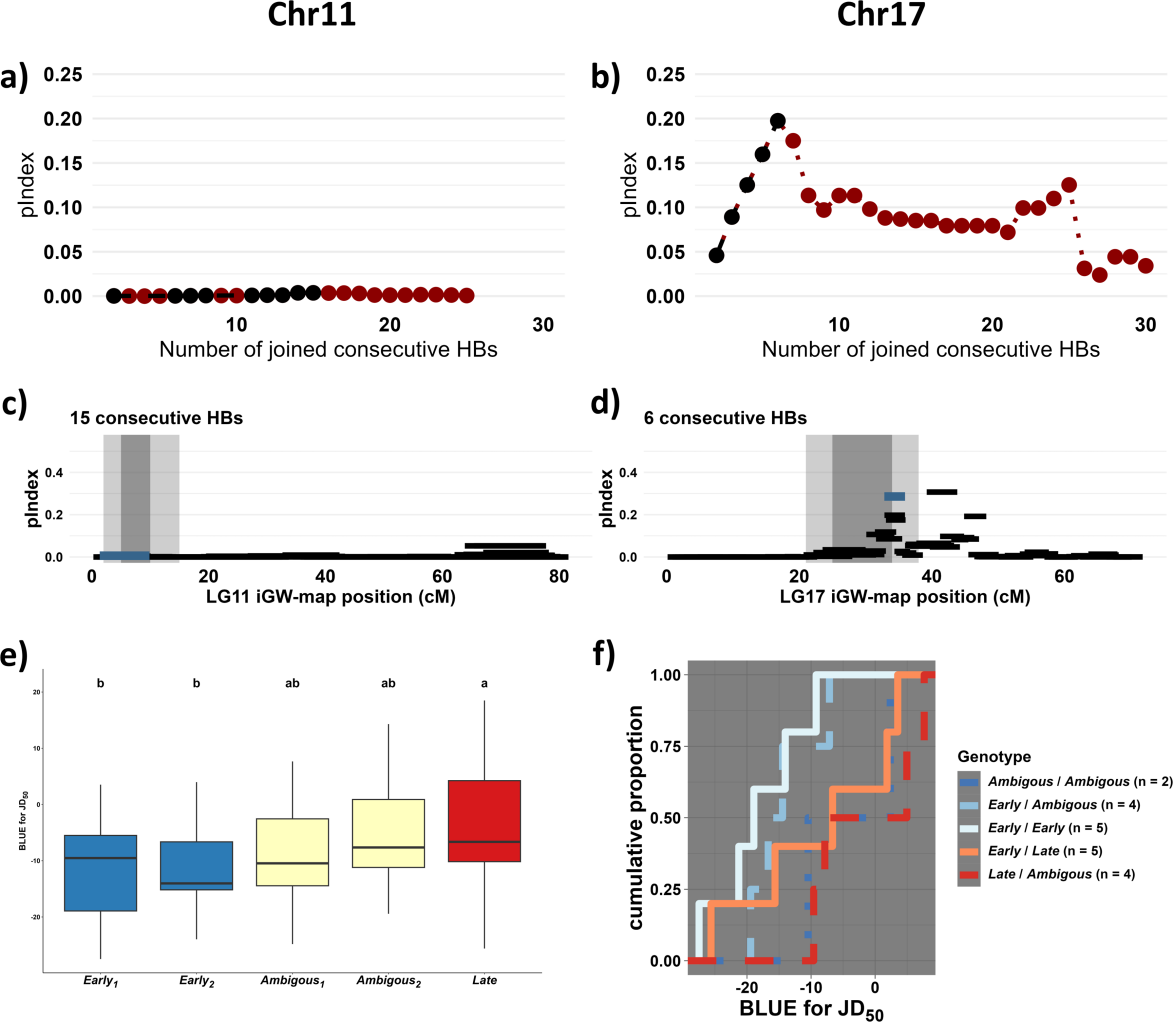
Haplotype association mapping in diversity germplasm, for chromosome 11 (a, c) and 17 (b, d–f), showing (a, b) development of pIndex for models with two to 30 consecutive haploblocks (HBs), (c, d) chromosome‐wide pancake plots for the most sensible number of joined consecutive HBs, (e) boxplot of individual haplotypes with Duncan‐groups indicated, and (f) the distribution of effective genotypes identified for chromosome 17. In (a, b), black dots indicate increasing pIndex of models, while red dots indicate no‐nincreasing pIndex of models. In (c, d), the joint HB with the highest pIndex while also overlapping with the inner QTL interval is indicated by a blue bar on each chromosome with other joint HBs in black, whereas the inner and outer QTL regions are indicated as dark‐ and light‐shaded gray areas, respectively.

Having established two QTL intervals in the MPP, I used a subset of 75 diploid cultivars from the Swedish Central collection with phased marker data available to assess the variability of the two QTL in a wider germplasm. The genomic region on chromosome 11 corresponding to the LG11 QTL did not show any discernible effect in this germplasm (Figure 6a,c). On the other hand, the genomic regions on chromosome 17 corresponding to the LG17 locus showed a clear effect, as models with up to six joint HBs showed a near‐linear increase in pIndex (Figure 6b) and a marked increase in pIndex of the retained joint HBs (Figure 6d).

The retained joint HB haplotype coinciding with the LG17 locus consisted of six consecutive HBs partially overlapping with the QTL interval, spanning 11.2–12.5 Mb_HFTH1_ corresponding to 32.6–36.0 cM_iGW_. This region comprised five IBS haplotypes with MAF > 0.05 in the germplasm, representing half of the haplotypes in the collection. The residuals of the linear model did not deviate significantly from normality (*p* > 0.05) or homoscedasticity (*p* > 0.05). Notably, a single haplotype was significantly different from the model intercept (*p* < 0.01) with an estimated effect of 8.4 days, hereby referred to as the *Late* allele. The following Duncan test revealed two haplotypes associated with early senescence, *Early*_*1*_ and *Early*_*2*_, as well as two haplotypes that were not different from either the late or the early haplotypes, designated *Ambigous*_*1*_ and *Ambigous*_*2*_ (Figure 6e and Supplementary File 2). Only 10 individuals had fully inferred genotypes, with an estimated delay in reaching 50% canopy senescence (JD_50_) of 9.7 days for the heterozygous *Early*/*Late* individuals, compared to the homozygous *Early*/*Early* (Figure 6f). On chromosome 17, the only IBS haplotype associated with JD_50_ that corresponded to a QTL‐haplotype in the MPP was the *Late* haplotype allele, which had an effect and inheritance consistent with the *q* allele (late senescence) from ‘Filippa’ segregating in ‘Aroma’.

### 3.5 Signals of Selection and Predicted Genes

**FIGURE 7:**
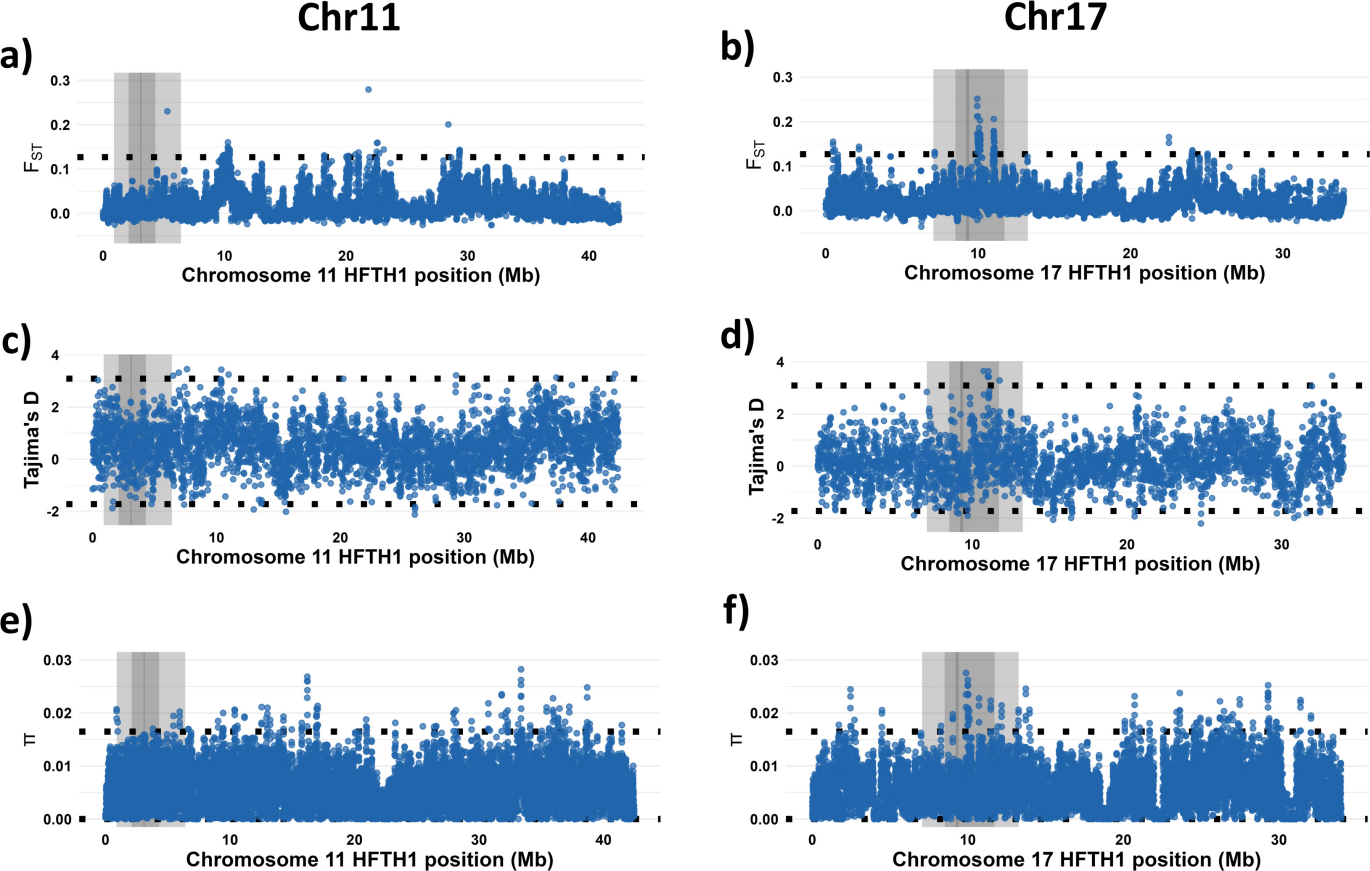
Chromosome‐wide patterns of signals of selection for chromosome (chr) 11 (a, c, e) and 17 (b, d, f). For each chromosome is shown (a, b) the weighted pairwise *F*_ST_ between the ‘hardy’ and ‘not hardy’ groups, (c, d) Tajima’s *D* in the ‘hardy’ group, and (e, f) nucleotide diversity (π) in the ‘hardy’ group. Genome‐wide 1% outlier thresholds are indicated as horizontal black‐dotted lines. For each chromosome, the genomic regions corresponding to the outer QTL interval, inner QTL interval, and QTL mode are indicated in light, medium, and dark gray, respectively. Note the drop in Tajimas *D* and π around 30 Mb_HFTH1_ of chromosome 17, near the S‐locus.

Next, I investigated the possibility of signals of selection among ‘hardy’ apple cultivars in the genomic regions on chromosomes 11 and 17 corresponding to the QTL intervals on LG11 and LG17. For chromosome 11, there were no outlier bins in *F*_ST_, Tajima’s *D*, or π in the genomic region corresponding to the inner QTL interval (Figure 7a,c,d), consistent with variation at this locus being rare in the wider Swedish germplasm. On the other hand, there were multiple bins on chromosome 17 with *F*_ST_, Tajima’s *D*, and π above the genome‐wide 1% outlier threshold (Figure 7b,d,f).

**FIGURE 8:**
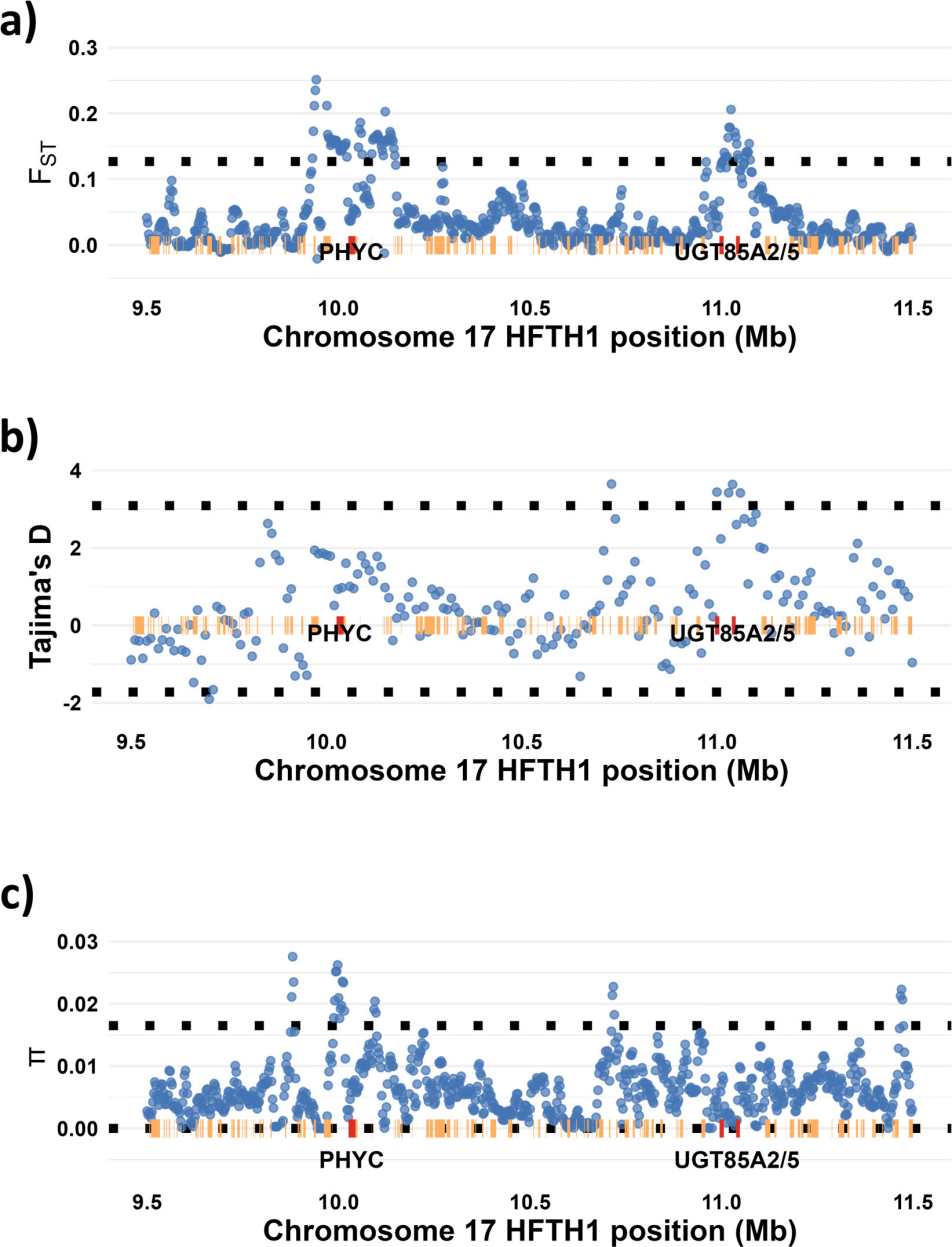
Local signals of selections in part of chromosome 17 corresponding to the inner LG17 QTL interval, showing (a) *F*_ST_, (b) Tajimas *D* in the ‘hardy’ group, and (c) nucleotide diversity (π) in the ‘hardy’ group. Genome‐wide 1% outlier thresholds are indicated as horizontal black‐dotted lines. Predicted genes are indicated as beige‐horizontal bars. Predicted genes with BLAST similarities to Arabidopsis PHYC and UGT85A2 are indicated by red bars, extended by 5 kb for visibility. Note that there are two PHYC copies around 10.04 Mb_HFTH1_, only 32 nt apart.

A closer examination revealed two candidate loci showing signals of selection within the genomic region corresponding to the inner QTL interval of the LG17 locus. For pairwise *F*_ST_ between groups, there were two outlier peaks, one centered on 10.04 Mb_HFTH1_ (corresponding to 29.33 cM_iGW_) and one around 11.04 Mb_HFTH1_ (corresponding to 32.14 cM_iGW_) (Figure 8a). The 11.04 Mb_HFTH1_ *F*_ST_ peak coincided with a peak in site frequency spectrum change (Tajimas *D*), but not the 10.04 Mb_HFTH1_ *F*_ST_ peak (Figure 8b). The 10.04 Mb_HFTH1_ *F*_ST_ peak coincided with a distinct local drop in nucleotide diversity (π), flanked by two peaks, while the 11.04 Mb_HFTH1_ *F*_ST_ peak coincided with a wider drop in nucleotide diversity (Figure 8c).

The peak in *F*_ST_ around 11.040 Mb_HFTH1_ coincided with two predicted genes, with annotated BLAST similarity to *Arabidopsis* UDP‐glucosyl transferase (UGT) 85A2 and 85A5 of the corresponding transcripts. The other peak in *F*_ST_, around 10.04 Mb_HFTH1_, was centered around two nearby predicted genes with annotated similarity to PHYC (phytochrome C, Figure 8), and close to the average mode of the LG17 QTL for %SEN (2.05 cM_iGW_ away).

Given the two very close putative PHYC copies at 11.040 Mb_HFTH1_ (only 32 nt apart), I reviewed the distribution of phytochromes across two haploid and 9 phased diploid *Malus* WGSs. Predicted genes with annotated similarities to PHYA, PHYB, and PHYE were distributed with typically one copy per haplotype of chromosomes 4 and 12, 13 and 16, and 5 and 10, following the pattern of the ancient duplication of the Pyreae genome (Velasco et al. 2010). In addition to chromosome 17, predicted genes with annotated similarity to PHYC were found on chromosome 9, as expected from the ancient genome duplication, and unexpectedly on 40% of the haplotypes of chromosome 7. Furthermore, around 1/3 of the haplotypes of chromosome 17 had one, two, and three predicted genes with annotated similarity to phytochromes, respectively, indicating a possible copy‐number variation among *M. domestica* cultivars. Notably, both haplotypes of both *M. sylvestris* and *M. sieversii* , the main wild progenitors of cultivated apple (Duan et al. 2017), had only one phytochrome copy on chromosome 17 and no copy on chromosome 7 (Table 3 and Supplementary File 2).

**TABLE 3:** Summary of distribution of predicted genes with annotated similarity to phytochrome encoding sequences among haplotypes of 11 whole‐genome sequences of *Malus*.

PHY copies	Chr04	Chr05	Chr07	Chr09	Chr10	Chr12	Chr13	Chr16	Chr17
0	—	0.05	0.6	0.1	0.05	0.1	0.1	0.05	0.05
1	1	0.95	0.4	0.85	0.95	0.9	0.85	0.95	0.35
2	—	—	—	0.05	—	—	0.05	—	0.3
3	—	—	—	—	—	—	—	—	0.25
4	—	—	—	—	—	—	—	—	0.05
Consensus^a^ homolog	PHYA	PHYE	PHYC	PHYC	PHYE	PHYA	PHYB	PHYB	PHYC

*Note:* There was no predicted gene with annotated similarity to PHYD on any WGS haplotype.

^a^ For Chr17, two of the 34 predicted genes were annotated as similar to PHYB, whereas the remaining were annotated as similar to PHYC.

## 4 Discussion

### 4.1 Autumn Senescence in the Swedish Central Collection of Heirloom Cultivars

The timing of different phenological growth stages of trees is expected to be crucial to their adaptation to temperate climates in general, and to boreal climate in particular. Here, I show that the timing of autumn canopy senescence is correlated with the adaptation of apple cultivars to different Swedish climate zones. Furthermore, I report a high broad sense heritability (*Ĥ* ^2^) and Year‐to‐ear correlation, in line with previous work on *Populus* (Richards et al. 2020). This implies that apple cultivars time their autumn senescence similarly over consecutive years at a specific location, that is, *Genotype* × *Year* interactions are small. This could be interpreted as an indication that photoperiod might serve as an environmental cue for the process, being stable across years. However, it might also be simply that the intrinsic *Genotype* effect is large. However, further work is needed to separate the effects of *Genotype*, *Environment*, and their interaction on autumn senescence, for example, by multi‐environmental trials or experiments under controlled conditions. However, I also report significant differences in the timing of autumn senescence between the 3 years and find this to be correlated with differences in temperature, expressed as CDDs below 15°C (CDD_15_). This indicates that temperatures below this threshold also affect this trait, but with very little interaction with genetic variation in the collection. Considering that climate change will result in altered temperatures, while photoperiods depend on latitude, a better understanding of how these environmental cues affect the autumn syndrome would be very valuable for further breeding. In *Populus* , growth cessation is mainly controlled by day length (Ding et al. 2024), whereas the timing of autumn senescence is not. A lack of clearly defined environmental cues for autumn senescence across locations in *Populus* has led to speculations about another, undefined, light‐dependent factor (Michelson et al. 2018). On the other hand, growth cessation in apple is generally reported to be insensitive to photoperiod (Ding et al. 2024; Heide and Prestrud 2005), with indications of genotype‐dependent interactions between photoperiod and temperature. Some authors have suggested two separate processes leading to dormancy induction in deciduous trees, which seem to be supported by global leaf senescence patterns of the Northern Hemisphere (Lang et al. 2024; Tanino et al. 2010). In short, either low temperatures or short days can function as environmental cues for dormancy induction, depending on genotype and climate. Recent work has indicated that PHYTOCHROME B, together with PHYTOCHROME INTERACTING FACTOR4 (PIF4), is involved in modulating growth cessation and bud set in *Populus* in response to both photoperiod and temperature (Zhang et al. 2025).

### 4.2 QTL Regions for Timing of Autumn Senescence

While I have presented decisive evidence for two QTL controlling the timing of autumn senescence in apple, it remains unclear if these QTL regulate autumn senescence per se or some other correlated component of the autumn syndrome. Autumn phenology traits are expected to be autocorrelated to some degree, as reported in *Populus* (Richards et al. 2020). In apple, leaf fall has been noted to coincide with the transition from endo‐ to ecodormancy in some genotypes (Milyaev et al. 2024). Regardless, a germplasm analysis revealed that the segregating variation on LG11 seems to be rare in both the MPP and in a wider diversity germplasm, while the LG17 locus seems to be variable in a wider germplasm of Swedish heirloom cultivars. Notably, the two alleles at LG17 associated with early senescence in the MPP (*Q*_Cox_ and *Q*_WP_, Figure 4) are IBS within the inner QTL interval (9 cM_iGW_). Being IBS over such a large interval, the two alleles could be IBD, through a probable close common ancestor for ‘Coxs Orange Pippin’ and ‘Worcester Pearmain’. These results clearly show that the approach for MPP QTL analysis inspired by previous work (Van de Weg et al. 2018) showed excellent power at mapping and post hoc analysis of QTL, even in the presence of dominance, epistasis, and segregation distortion. Being able to appreciate the genetic interactions affecting a trait provides valuable information for further breeding and research efforts, beyond the identification of a segregating locus.

Interestingly, two synergistic loci conferring quantitative resistance to apple scab ( *Venturia inaequalis* ) have previously been mapped to LG11 (0–21.0 cM_Bénéjam_) and 17 (14.2–27.3 cM_Bénéjam_) in segregating offspring of ‘Fiesta’ (Bénéjam et al. 2021). While genetic map positions might not be fully comparable between studies, these QTL intervals for scab resistance could indeed overlap with the hereby presented loci for canopy autumn senescence. The cultivar ‘Fiesta’ is an offspring of ‘Coxs Orange Pippin’ and seems to have inherited the *Q*_Cox_ allele segregating in the MPP IBD within the inner QTL interval at LG17 (not shown). Also, the LG11 haplotype of ‘Fiesta’ coming from ‘Coxs Orange Pippin’ appears to be IBS within the inner QTL interval with the *Q* allele from ‘Coxs Pomona’ segregating in ‘Aroma’ (not shown). It is also worth noting that *V. inaequalis* appears to exhibit a strictly biotrophic lifestyle (Steiner and Oerke 2024). Plant resistance mechanisms against biotrophic fungi typically involve processes similar to senescence, while the pathogen attempts to delay senescence (Häffner et al. 2015). However, further work would be needed to confirm if either of the two described QTL for quantitative scab resistance and canopy autumn senescence are indeed caused by the same loci.

A potentially epistatic candidate locus was also identified on LG9, partially overlapping with a previously described QTL for bud break in apple (0–10 cM_draft‐iGLMap_, Allard et al. 2016). While a homolog of *Arabidopsis AGL24* was highlighted as a candidate gene in that study, the genomic region corresponding to the current QTL interval also contains a predicted gene with annotated similarity to a PHYC encoding sequence. The candidate QTL had a mode at 18 cM_iGW_, corresponding to approximately 6 Mb_HFTH1_, and the HFTH1 WGS has one predicted gene with annotated similarity to the transcript of AGL42 at 6.7 Mb_HFTH1_. On the other hand, the predicted PHYC encoding gene is located at 9.5 Mb_HFTH1_, thus being further from the mode but well within the QTL interval. Genomic regions associated with flowering period have also been identified at the top of chromosome 9 (two SNPs at 0.53 and 0.56 Mb_GDDH13_, resp., Urrestarazu et al. 2017), and a QTL for chill requirement during endodormancy has been mapped to the top of LG9 (0.44–10.48 Mb_GDDH13_, Cornelissen et al. 2020). Thus, the putative epistatic candidate locus for autumn senescence on LG9 could be caused by a unique locus, linked to the other loci associated with other phenology traits, or by pleiotropic effects of one of the previously described loci.

### 4.3 Candidate Genes Under Selection

The genomic region on chromosome 11 corresponding to the inner QTL interval showed no signals of selection among the ‘hardy’ apple cultivars, consistent with variability at this locus being rare in a wider germplasm. On the other hand, I identified two loci with indications of differential selection between ‘hardy’ and ‘not hardy’’ apple cultivars coinciding with the LG17 locus. Although the locus around 10.040 Mb_HFTH1_ coincided with a distinct drop in nucleotide diversity, possibly indicating a recent hard sweep, the locus around 11.040 Mb_HFTH1_ coincided with a positive peak in Tajima’s *D*. Positive values of Tajima’s *D* (i.e., an excess of alleles at intermediate frequency) are usually taken to indicate population structure or balancing selection, but can also be generated by a partial sweep where the favorable allele is currently undergoing selection and increasing in frequency in the population (Walsh and Lynch 2018). Apple cultivation spread to northern Sweden relatively late, such that the first apples in the province Västerbotten (eng. “West Bothnia”) are supposed to have been harvested in the town Skellefteå (app. 100 km north‐east of Umeå, Figure 1) in 1771 (Nilsson 1987). Considering the long generation and lifetime of apple, selection for alleles that are favorable for adaptation to higher latitudes would indeed have started very recently.

Considering candidate genes, the region at 11.040 Mb_HFTH1_ comprised two predicted genes with annotated similarity to *Arabidopsis* uridine diphosphate (UDP) glycosyltransferases (UGTs). In *Arabidopsis*, *UGT85A5* is expressed mainly in mature leaves while *UGT85A2* is expressed in petioles of senescent leaves (Klepikova et al. 2016). UGTs have also been reported to be associated with leaf senescence in cotton (Chen et al. 2022), though plant UGTs constitute a large gene family involved in many processes.

The region at 10.040 Mb_HFTH1_ is centered over two predicted genes with annotated similarity to phytochrome C encoding sequences (*PHYC*) of *Arabidopsis*. Notably, a copy number variation seems to exist among the published *Malus* WGSs assessed. The WGSs of the wild progenitors of cultivated apple, *M. sieversii* and *M. sylvestris* , had only one copy each per haplotype, which could indicate that this variation is new and not originating from either of the two parental species. Phytochromes are mainly associated with the perception of red and far‐red light signals, but can also function as thermal sensors (Jung et al. 2016; Legris et al. 2019). The regulatory networks controlling vegetative growth in *Populus* show major similarities to that controlling floral initiation in *Arabidopsis* , with PHYA and PHYB and PtPHYA, PtPHYB1, and PtPHYB2 as the main photoreceptors in *Arabidopsis* and *Populus* , respectively (Ding and Nilsson 2016). However, PHYC has also been shown to play a role in daylength perception in *Arabidopsis* (Monte et al. 2003; Sánchez‐Lamas et al. 2016), and variation in *PHYC* has been found to account for a large proportion of the variation in flowering time across European latitudes (Balasubramanian et al. 2006; Lempe et al. 2005). However, considering the above‐discussed potential overlap with QTL for quantitative scab resistance, the UGTs at 11.040 Mb_HFTH1_ would be more likely to be involved in both processes than the PHYC copies at 10.040 Mb_HFTH1_, if the processes are indeed connected.

## 5 Conclusions

Understanding the genetic factors controlling climate adaptation is fundamental to an efficient targeted breeding approach and the mitigation of climate change. However, plant adaptation to a specific climate is complex and likely to depend upon several specific traits. By combining historical data, phenotypic screening of a diversity germplasm, MPP QTL mapping, and a scan for genomic signals of selection, I here provide data that support early autumn senescence as an adaptive trait for northern Sweden, identify QTL alleles for variation, and describe candidate genes showing signals of recent selection. However, further work is needed to fully describe the segregating alleles and to better understand how autumn senescence is controlled in a germplasm more closely related to the Swedish heirloom cultivars. The interaction between autumn senescence and other traits involved in the autumn syndrome also has to be further explored to verify whether the described loci are specific for autumn senescence or shared with other autumn phenology traits. Further work is also needed to assess the interaction between temperature, light, and genotype with regard to autumn phenology in apple. This has important implications for the way plant breeding can mitigate the ongoing climate change, with changing temperatures but fixed photoperiods.

## Author Contributions

Jonas Skytte af Sätra conceived the study, performed the phenotyping, analyzed the data, and wrote the manuscript.

## Conflicts of Interest

The author declares no conflicts of interest.

## Supporting information


**Figure S1:** The location of Alnarp and Balsgård in Sweden (a), and the orientation of the Swedish Central collection (b) and the multiparental populations (c). The different years of planting for most of the trees in each section are indicated in (b), whereas the multiparental populations are indicated in black and the approximate position of the outlier trees excluded from analysis are indicated in purple in (c). Modified from google maps [accessed 2025‐05‐29].
**Figure S2:** Daily mean temperatures at the Lönnstorp field station in August–December, 2019–2021. Relative Julian dates corresponding to year effects plus intercept are indicated as triangles for each year.
**Figure S3:** Daily precipitation at the Lönnstorp field station in August–December, 2019–2021. Relative Julian dates corresponding to year effects plus intercept are indicated as triangles for each year.
**Figure S4:** Daily incoming shortwave radiation at the Lönnstorp field station in August–December, 2019–2021. Relative Julian dates corresponding to year effects plus intercept are indicated as triangles for each year.
**Figure S5:** Correlation between JD50 and *r* for each individual tree in each year (*n* = 1178), with a linear trendline indicated as a dark red dashed line and 95% confidence interval indicated as shaded red.
**Figure S6:** Trace plots from four FlexQTL runs.
**Figure S7:** Frequency of SNP alleles with paternal monohybrid segregation for alleles from ‘Ingrid Marie’ and ‘James Grieve’, respectively, on LG17, exhibiting segregation distortion, and LG11 for reference. Alleles that are common to both parents are indicated in blue, whereas alleles that are unique to the pollen parent are indicated in red. The A and B alleles are indicated as circles and triangles, respectively. Inner QTL intervals and S‐locus are indicated as shaded gray areas.

**Table S1:** Cultivars. A list of cultivars in the Swedish Central collection sensu Skytte af Sätra et al. (2020), analyzed in Skytte af Sätra et al. (2024), or shown as ancestors in Figure 4.
**Table S2:** Summary of highest and lowest LG‐wide 2lnBF for the number of QTL per LG, across four replicate runs of FlexQTL. A 2lnBF value above 2, 5, and 10 indicates positive, strong, and decisive evidence for the number of QTL per LG. 2lnBF values above 10 are indicated in bold.
**Table S3:** Parental haplotypes of the outer QTL intervals segregating in the multiparental populations, considering the pruned set of SNP markers used for QTL mapping. The haplotype of the *q* alleles are indicated by a lightgray background. The average QTL mode is also indicated, with functional allele assignment instead of 20K SNP array genotypic data.
**Table S4:** Germplasm haplotypes in the Swedish Central collection associated with JD_50_.
**Table S5:** Summary of predicted genes with annotated BLAST similarity to phytochromes of the corresponding transcripts in the GDR.

## Data Availability

BLUEs for JD_50_ for cultivars in the Swedish Central collection, phased SNP calls of the parents of the MPP of each QTL interval, and phased SNP calls of IBS haplotypes associated with JD_50_ are given in Supplementary File 2. The whole‐genome sequencing raw reads used in (Skytte af Sätra et al. 2024) have previously been deposited in ENA under project accession PRJEB71391.
