# Supplementary material for: Genetic Analysis of Apple Autumn Canopy Senescence in a Nordic Climate
Source: Physiol Plant. 2025 Oct 30;177(6):e70599. doi: 10.1111/ppl.70599 (PMC12573221; doi:10.1111/ppl.70599)
Supplement: Supplementary file 1 — Figure S1: The location of Alnarp and Balsgård in Sweden (a), and the orientation of the Swedish Central collection (b) and the multiparental populations (c). The different years of planting for most of the trees in each section are indicated in (b), whereas the multiparental populations are indicated in black and the approximate position of the outlier trees excluded from analysis are indicated in purple in (c). Modified from google maps [accessed 2025‐05‐29]. Figure S2: Daily mean temperatures at the Lönnstorp field station in August–December, 2019–2021. Relative Julian dates corresponding to year effects plus intercept are indicated as triangles for each year. Figure S3: Daily precipitation at the Lönnstorp field station in August–December, 2019–2021. Relative Julian dates corresponding to year effects plus intercept are indicated as triangles for each year. Figure S4: Daily incoming shortwave radiation at the Lönnstorp field station in August–December, 2019–2021. Relative Julian dates corresponding to year effects plus intercept are indicated as triangles for each year. Figure S5: Correlation between JD50 and r for each individual tree in each year (n = 1178), with a linear trendline indicated as a dark red dashed line and 95% confidence interval indicated as shaded red. Figure S6: Trace plots from four FlexQTL runs. Figure S7: Frequency of SNP alleles with paternal monohybrid segregation for alleles from ‘Ingrid Marie’ and ‘James Grieve’, respectively, on LG17, exhibiting segregation distortion, and LG11 for reference. Alleles that are common to both parents are indicated in blue, whereas alleles that are unique to the pollen parent are indicated in red. The A and B alleles are indicated as circles and triangles, respectively. Inner QTL intervals and S‐locus are indicated as shaded gray areas. [file PPL-177-e70599-s001.docx]

**Supplementary File 1.**

Skytte af Sätra J^1^* (2025) *Genetic analysis of apple autumn canopy senescence in a Nordic climate*, Physiologia Plantarum

^1^Department of Plant Breeding, Swedish University of Agricultural Sciences, Alnarp, Sweden

*Corresponding author: jonas.skytte.af.satra@slu.se

**Table of content**

Fig.1 Maps.

Fig.2 Daily mean temperatures.

Fig.3 Daily precipitation.

Fig.4 Daily shortwave radiation.

Fig.5 Correlation between JD_50_ and *r*.

Fig.6 FlexQTL^TM^ trace plots.

Fig.7 Paternal segregation distortion.


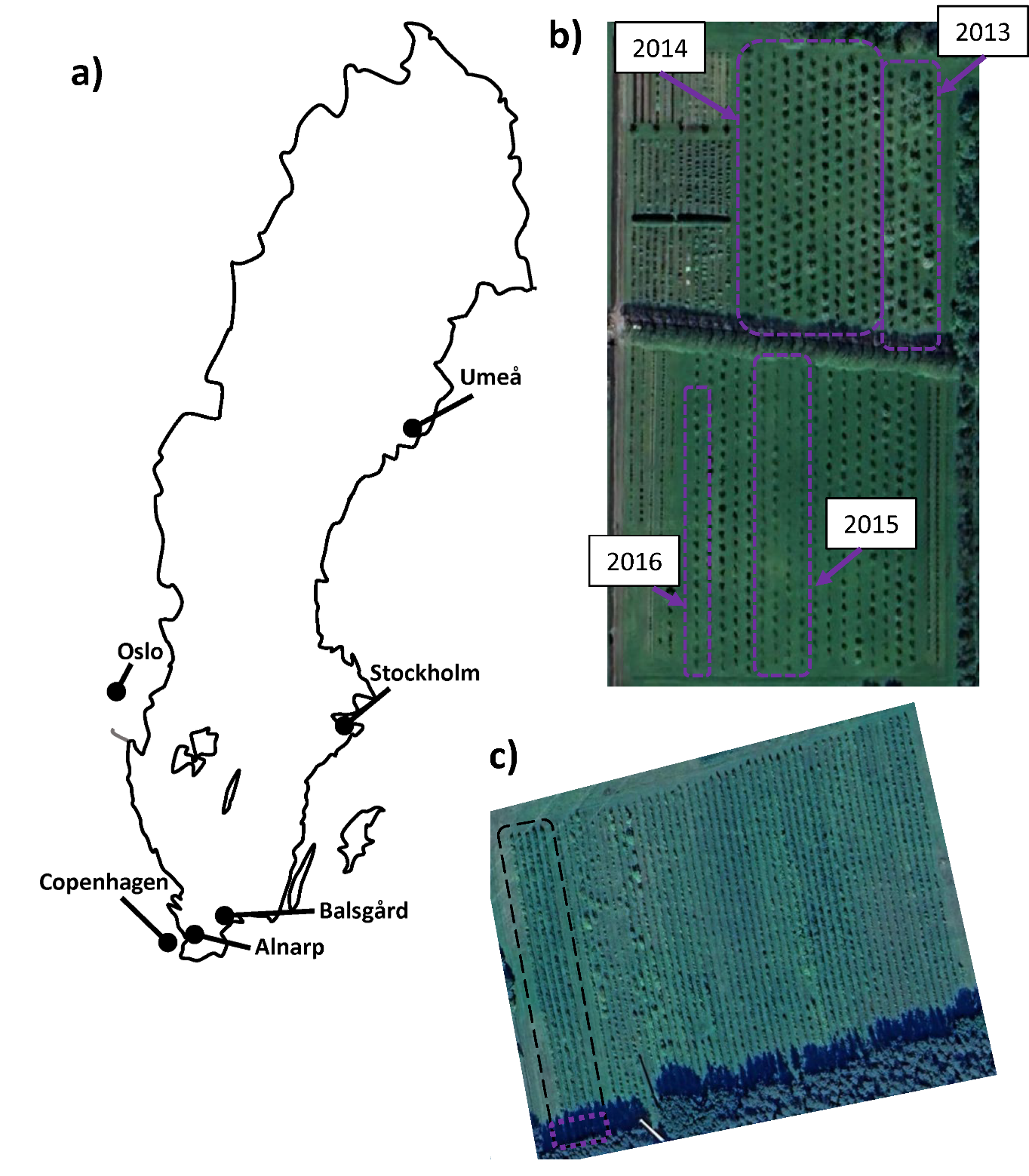


*Figure 1. The location of Alnarp and Balsgård in Sweden (a), and the orientation of the Swedish Central collection (b) and the multiparental populations (c). The different years of planting for most of the trees in each section are indicated in (b), while the multiparental populations are indicated in black and the approximate position of the outlier trees excluded from analysis are indicated in purple in (c). Modified from google maps [accessed 2025-05-29].*

*
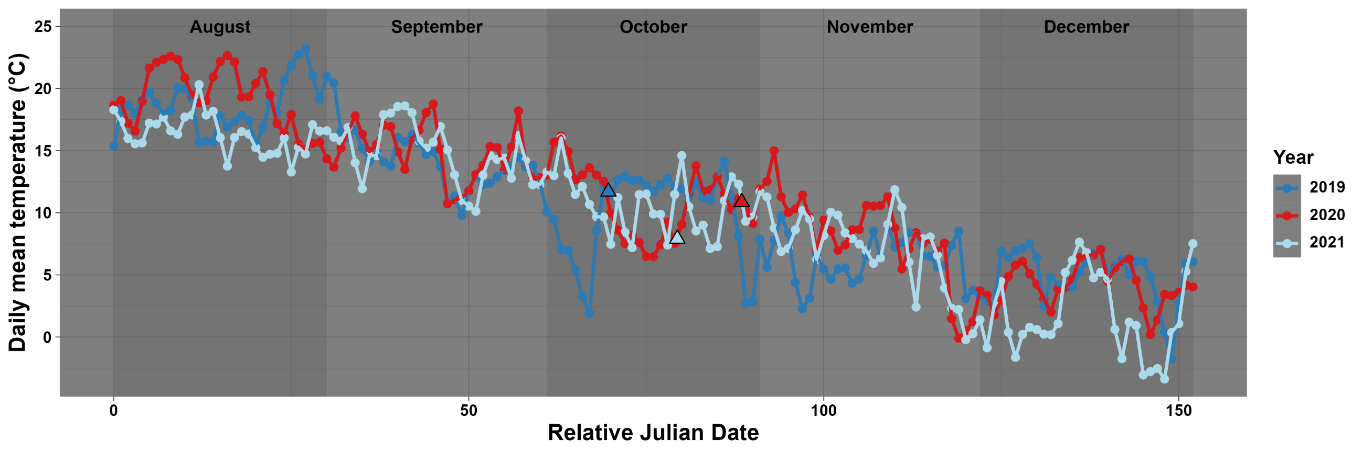
*

*Figure 2. Daily mean temperatures at the Lönnstorp field station in August – December, 2019 – 2021. Relative Julian Dates corresponding to Year effects plus intercept are indicated as triangles for each year.*


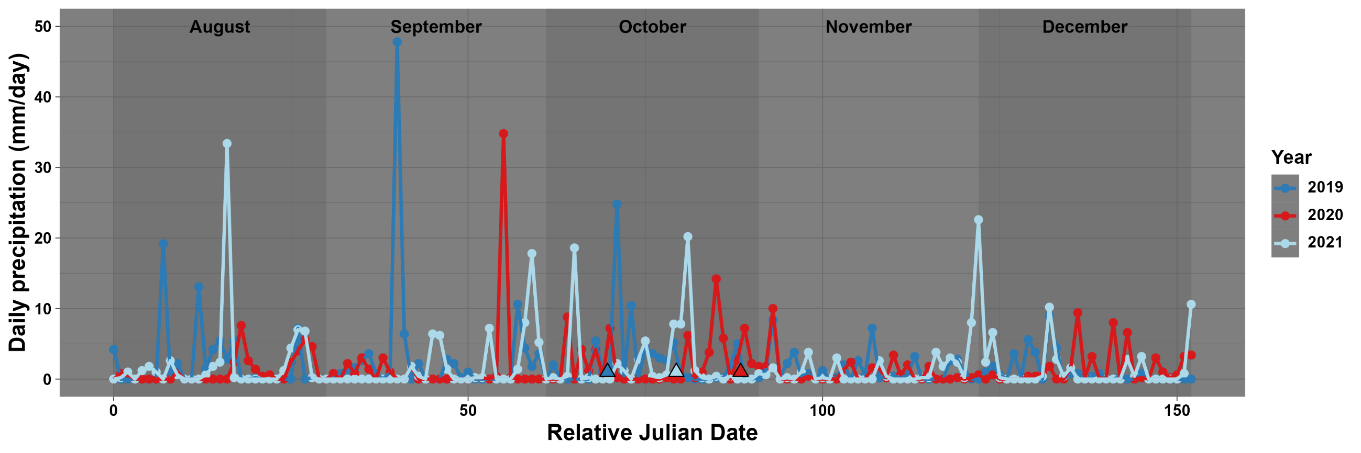


*Figure 3. Daily precipitation at the Lönnstorp field station in August – December, 2019 – 2021. Relative Julian Dates corresponding to Year effects plus intercept are indicated as triangles for each year.*


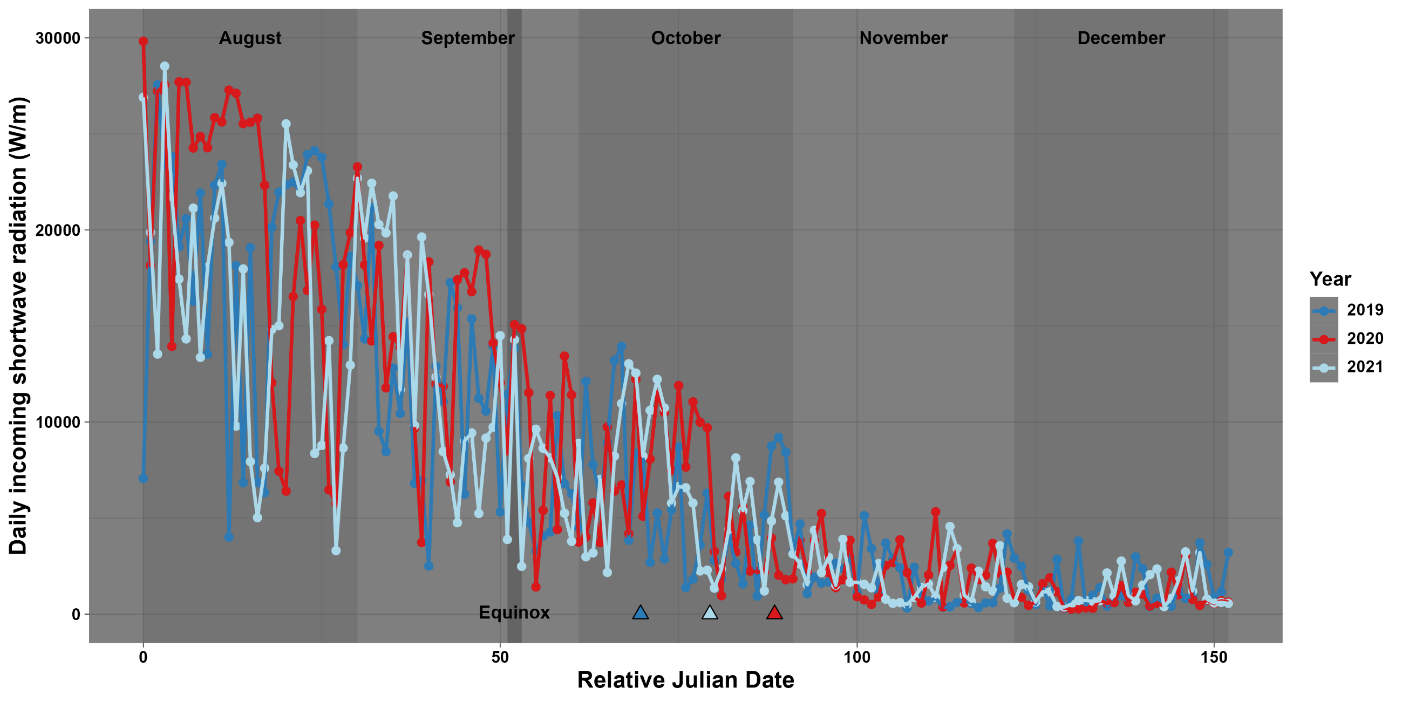


*Figure 4. Daily incoming shortwave radiation at the Lönnstorp field station in August – December, 2019 – 2021. Relative Julian Dates corresponding to Year effects plus intercept are indicated as triangles for each year.*


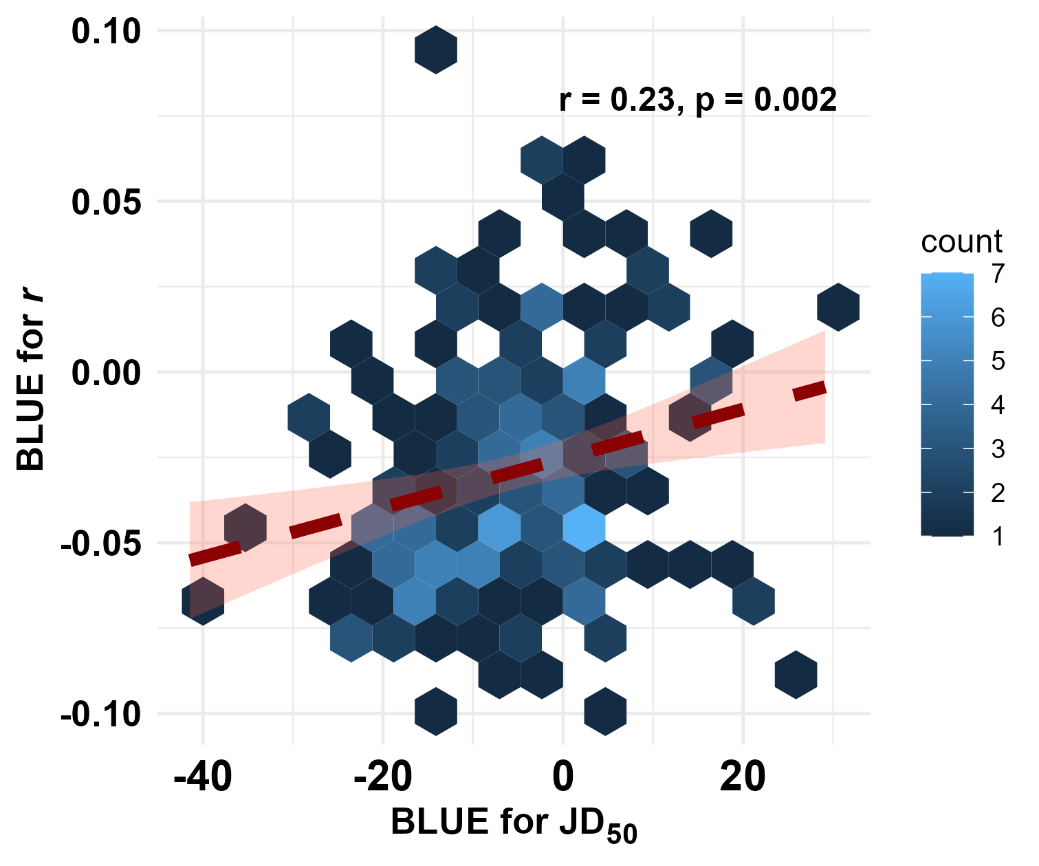


*Figure 5. Correlation between JD_50_ and r for each individual tree in each year (n = 1,178), with a linear trendline indicated as a dark red dashed line and 95% confidence interval indicated as shaded red.*

*
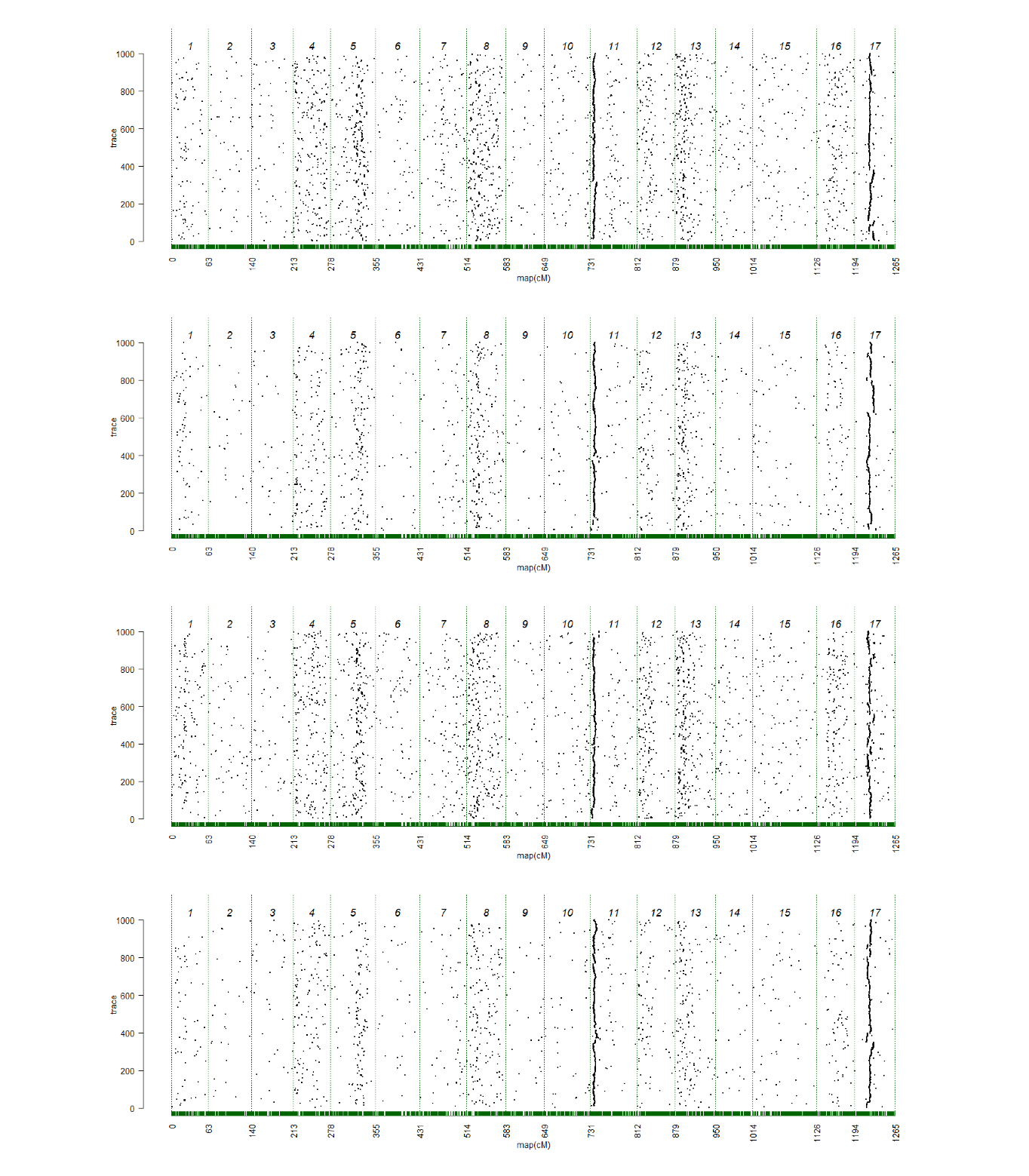
*

*Figure 6. Trace plots from four FlexQTL^TM^ runs.*

*
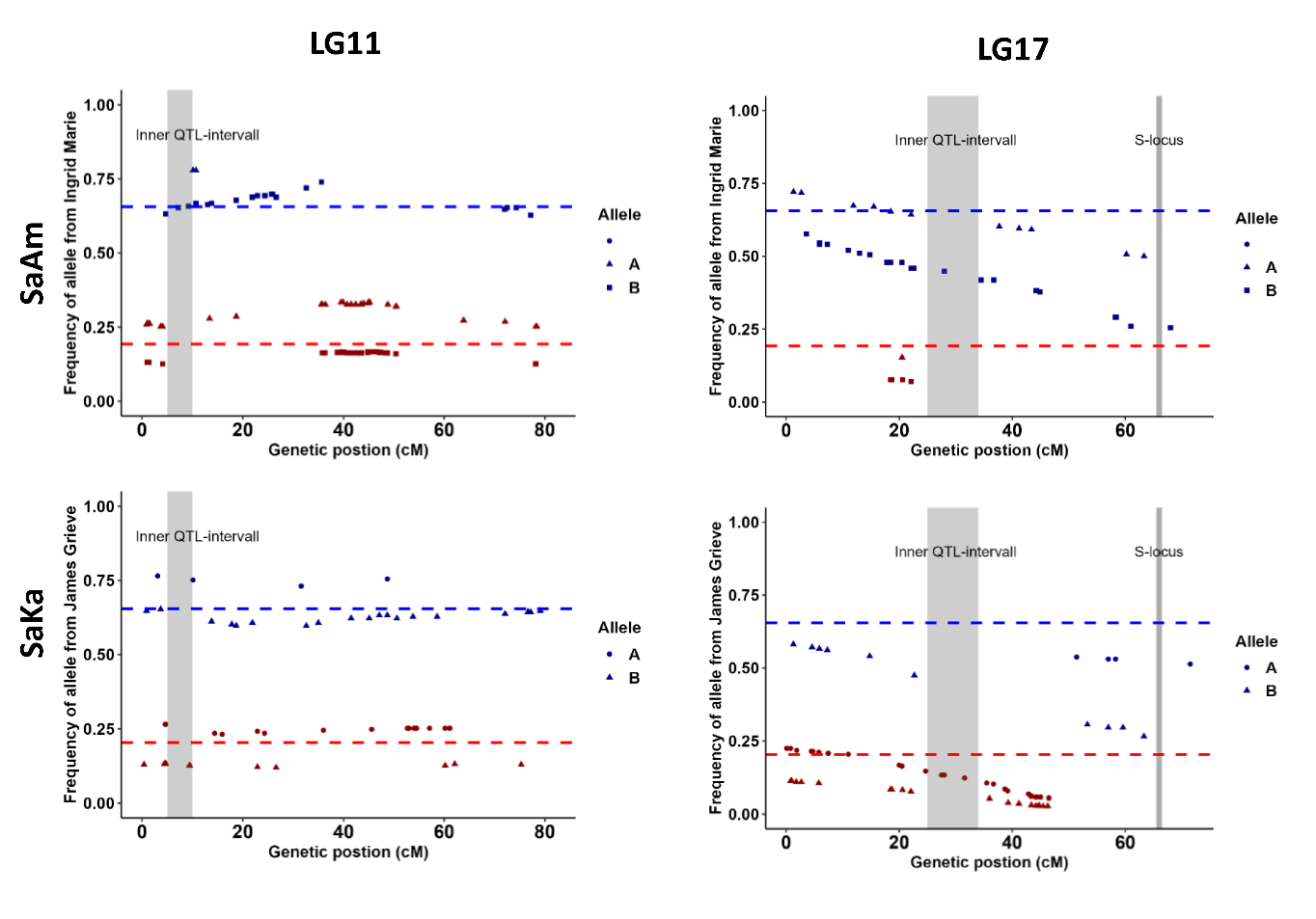
*

*Figure 6. Frequency of SNP alleles with paternal monohybrid segregation for alleles from ‘Ingrid Marie’ and ‘James Grieve’, respectively, on LG17, exhibiting segregation distortion, and LG11 for reference. Alleles that are common to both parents are indicated in blue, while alleles that are unique to the pollen parent are indicated in red. The A and B alleles are indicated as circles and triangles, respectively. Inner QTL intervals and S-locus are indicated as shaded grey areas.*
